# Organic Cage Rotaxanes

**DOI:** 10.1002/chem.202501014

**Published:** 2025-05-27

**Authors:** Zarik Zaheer Yusaf, Benjamin D. Egleston, Gokay Avci, Kim E. Jelfs, James E. M. Lewis, Rebecca L. Greenaway

**Affiliations:** ^1^ Department of Chemistry, Imperial College London Molecular Sciences Research Hub White City Campus, Wood Lane London W12 0BZ UK; ^2^ School of Chemistry University of Birmingham, Molecular Sciences Building Edgbaston Birmingham B15 2TT UK

**Keywords:** CuAAC, interlocked, organic cages, porous materials, rotaxanes

## Abstract

Organic cages are a robust class of molecular hosts with a myriad of applications in materials science. Despite this, there has been a paucity of explorations into the modification of their properties via external functionalization. In this work, [*n*]rotaxanes featuring unoccupied organic cages as stopper components and a small 2,2′‐bipyridine macrocycle were constructed using the active metal template (AMT) approach. By exploiting a scrambling methodology, it was possible to synthesize cages with a defined number of interlocked components (*n* = 2–4). The gas uptake, solubility, and thermal properties of the interlocked systems were compared against those of their constituent, non‐interlocked components. In this manner, we were able to demonstrate the potential of exploiting the mechanical bond for modulating the physiochemical properties of these molecular materials.

## Graphical abstract

The synthesis of robust, amine‐based organic cages with a controllable number of alkyne functional groups is described. Subsequent post‐synthetic modification enabled installation of a defined number of rotaxane units on the external surfaces, resulting in changes to the physiochemical properties of the cages.

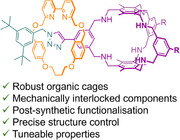

## 1 Introduction

Organic cages are discrete molecular systems assembled through covalent bond formation and possessing internal cavities capable of binding guest species.^[^ ^1^, ^2^ ^]^ Shape‐persistent cages with a permanent accessible cavity are often referred to as porous organic cages (POCs).^[^ ^3^, ^4^, ^5^, ^6^ ^]^ The ability of POCs to encapsulate guests has led to their use in a variety of applications including catalysis,^[^ ^7^ ^]^ gas sorption,^[^ ^8^ ^]^ and molecular separations.^[^ ^9^ ^]^ Furthermore, the synthetic tunability of organic cages^[^ ^10^ ^]^ has allowed a wide range of topologies to be realized and for them to be studied as materials in the solid state, in solution, and as porous liquids.^[^ ^11^ ^]^

Dynamic covalent chemistry (DCC),^[^ ^12^ ^]^ such as imine bond formation, is often exploited for the synthesis of POCs due to the “error correction” inherent to reversible chemical bonds, enabling access to the most thermodynamically favorable structures in high yields. The modularity of DCC also permits facile exchange of related building blocks to modulate the properties of organic cages. In 2011, for example, Cooper and coworkers reported the tunable porosity of a “scrambled” mixture of POCs assembled from a trialdehyde and a combination of two diamine building blocks, compared to topologically isostructural POCs formed from just a single diamine, due to increased structural disorder.^[^ ^13^ ^]^ Later, this group also reported the increased solubility of a similar, structurally analogous scrambled mixture of POCs due to increased structural disorder.^[^ ^11^, ^14^ ^]^

While the dynamic nature of imine bonds makes them highly convenient for preparing POCs, they can be hydrolytically unstable, although there are exceptions,^[^ ^15^, ^16^, ^17^ ^]^ limiting the utility of the cages. To overcome this, imines can be “fixed” by reduction to amines,^[^ ^18^, ^19^, ^20^ ^]^ providing a level of kinetic robustness, although sometimes at the expense of their permanent porosity due to structural collapse resulting from increased flexibility.^[^ ^21^ ^]^ “Fixing” can also allow post‐synthetic modification (PSM)^[^ ^22^ ^]^ of the cage scaffolds to both modify their properties and install functional units ^[^ ^23^ ^]^ that might otherwise be incompatible with dynamic covalent bonds.

There have been a number of previous reports of POCs that, rather than forming as monomeric architectures, undergo interpenetration to form mechanically interlocked molecules (MIMs).^[^ ^24^, ^25^, ^26^, ^27^, ^28^ ^]^ Often this interlocking significantly reduces the accessibility of the internal cavity space, limiting guest‐binding potential. The incorporation of mechanically interlocked components on the periphery of the POCs could, however, be used to modify the structure and thus properties of the cages while theoretically leaving the cavity intact. Indeed, mechanically interlocked molecule (MIM) architectures have previously been used to modulate the solubility,^[^ ^29^, ^30^ ^]^ electronic,^[^ ^31^, ^32^ ^]^ and biological properties^[^ ^33^, ^34^, ^35^, ^36^ ^]^ of molecular components. Although there have been reports of coordination cages functionalized with interlocked components,^[^ ^37^, ^38^ ^]^ the introduction of MIMs onto organic cages has yet to be explored.

In this work, we present the synthesis of organic cage[*n*]rotaxanes by exploiting the copper(I)‐catalyzed azide‐alkyne cycloaddition (CuAAC) active metal template (AMT) approach.^[^ ^39^, ^40^ ^]^ Using this method, we were able to mechanically append macrocyclic components to peripherally functionalize organic cages with unoccupied cavities. Subsequent investigations into the differences in physiochemical properties between the interlocked and non‐interlocked species demonstrated the potential for using the mechanical bond to modify the behavior of these discrete systems.

## 2 Results and Discussion

**Figure 1:**
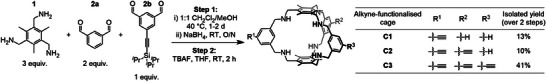
Synthesis of alkyne‐functionalized cages **C1**‐**C3** through imine condensation between triamine **1** and dialdehydes **2**, followed by reduction.

For our core structure, we chose a Tri^2^Di^3^ or [2+3] cage design (where for Tri*^x^* Di*^y^* or [*x* + *y*], *x*  = number of tritopic building blocks and *y*  = number of ditopic building blocks in the cage structure), originally reported by Delgado and coworkers.^[^ ^41^ ^]^ This system was formed through imine condensation between triamine **1** and dialdehyde **2a**, followed by reduction to give the robust amine cage **C** (Figure 1). To incorporate interlocked macrocycle components via CuAAC‐AMT, alkyne units were chosen to be installed on the periphery of the structure. To this end, an analogous dialdehyde, **2b**, was synthesized incorporating a triisopropylsilyl (TIPS)‐protected alkyne. To allow control over the incorporation of a defined number of mechanically interlocked components, which would enable us to investigate how the extent of functionalization affected the properties of the cage, we sought to generate POCs with one to three alkyne units on the periphery (**C1** to **C3**, respectively). To achieve this, a scrambled library of DCC cages^[^ ^13^, ^42^ ^]^ was generated through the reaction of triamine **1** with a mixture of dialdehydes **2a** and **2b** in a 3:2:1 ratio (Figure 1). An excess of triamine **1** was used as this has previously been shown to promote conversion to the targeted cages.^[^ ^43^ ^]^

The imine cages were then reduced in situ to form a mixture of amine organic cages that could be separated by column chromatography, yielding the cages **C1^TIPS^** and **C2^TIPS^** in 20% and 14% isolated yield, respectively. Removal of the TIPS protecting groups furnished the terminal alkyne‐functionalized cages, **C1** and **C2**, with one and two alkyne units, respectively. The tri‐alkyne cage **C3** was directly synthesized using three equivalents of dialdehyde **2b** and triamine **1**, followed by reduction and deprotection, in 41% overall yield. The successful synthesis of each of **C1**‐**C3** was confirmed by high‐resolution electrospray ionization mass spectrometry (HR‐ESI‐MS) and nuclear magnetic resonance (NMR) spectroscopy.

**Figure 2:**
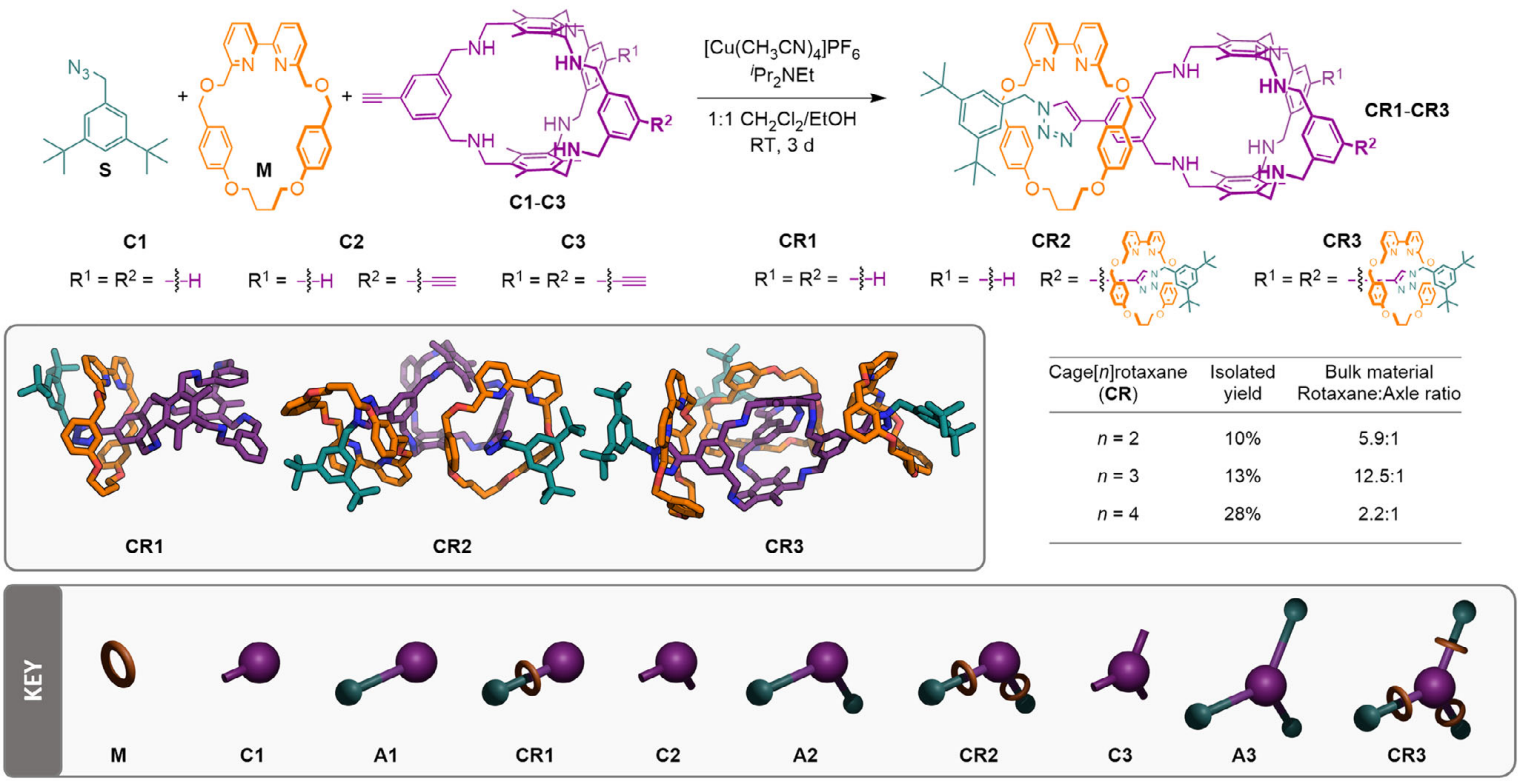
Synthesis of cage[*n*]rotaxanes (**CR1‐3**) using the CuAAC‐AMT methodology. Inset: Energy‐minimized molecular models of the rotaxane structures (hydrogens not shown for clarity) and a generalized key for the different structures included in this study.

With the alkyne‐functionalized cages in‐hand, the synthesis of cage[*n*]rotaxanes was envisaged using Goldup and coworkers’ small 2,2′‐bipyridine macrocycle modification^[^ ^44^ ^]^ of Leigh and coworkers’ CuAAC‐AMT method^[^ ^45^, ^46^ ^]^ with macrocycle **M** ^[^ ^47^ ^]^ and 3,5‐di‐*tert*‐butylbenzyl azide, **S**, as a stopper (Figure 2). Molecular modelling (molecular dynamics in Macromodel, OPLS4 forcefield)^[^ ^48^ ^]^ of each of the prospective rotaxane structures confirmed a loss in shape‐persistency of the cage core on reduction of the imines, as expected, but indicated that the cage framework should be large enough to prevent dethreading of **M** (as illustrated in Fig. S209).

**Figure 3:**
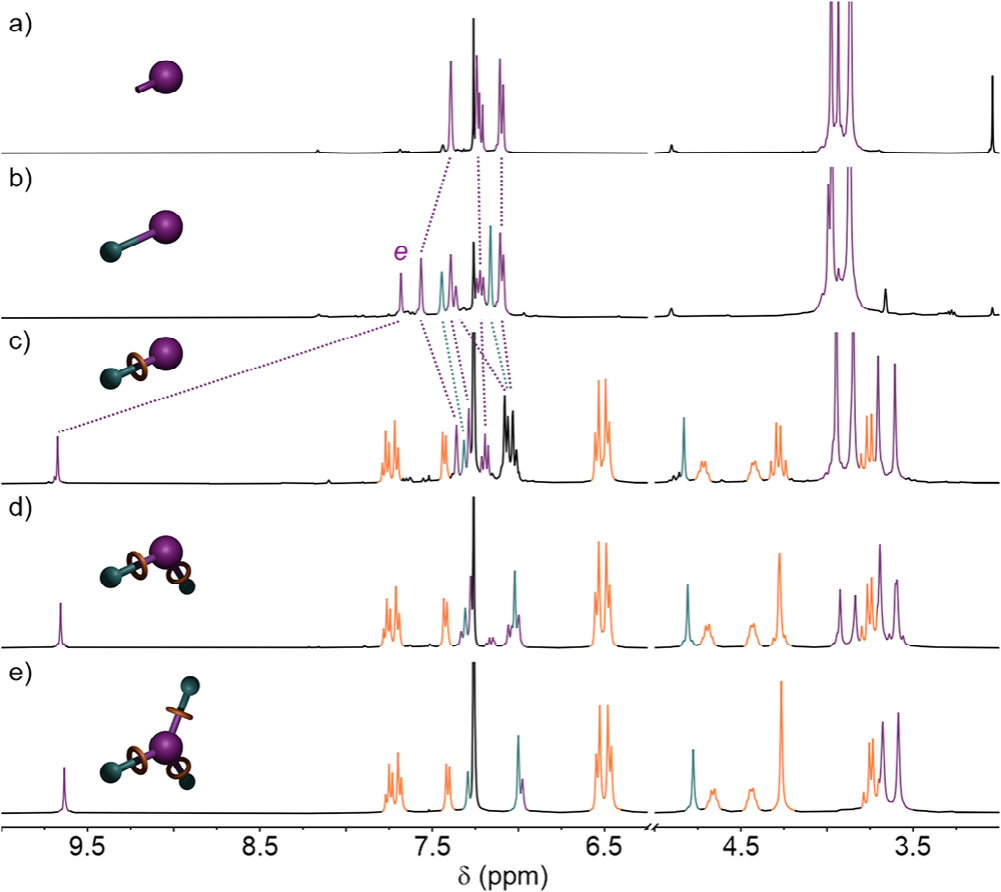
Partial ^1^H NMR spectra (400 MHz, CDCl_3_, 298 K) of a) **C1**, b) **A1**, c) **CR1**, d) **CR2**, and e) **CR3**.

Initially, we confirmed the viable reactivity of the alkyne units through reaction of **C1**‐**C3** with **S** under standard CuAAC conditions (CuSO_4_·5H_2_O, sodium ascorbate, DMF, RT). Pleasingly, each of the non‐interlocked axle cages with 1–3 triazole units (**A1**‐**A3**, respectively), was obtained in good isolated yield (38‐64%). Subsequently, each of **C1**‐**C3** was submitted to CuAAC‐AMT conditions which, following column chromatography, yielded the target [2]‐, [3]‐, and [4]‐rotaxane structures (**CR1**, **CR2,** and **CR3**, respectively), in quantities >100 mg. This bulk material was contaminated with small amounts of non‐interlocked triazole components that could be quantified by ^1^H NMR spectroscopy (Figure 3). Due to difficulties in separating the interlocked and non‐interlocked components by standard chromatographic techniques, isolation of the pure rotaxanes **CR1**, **CR2,** and **CR3** required reverse‐phase preparative TLC, giving the interlocked structures in isolated yields of 10%, 13%, and 28%, respectively. Such purification techniques, however, limited the amount of pure **CR** products that could be brought through (<30 mg).

Successful rotaxane formation was confirmed by HR‐ESI‐MS and NMR spectroscopy (Figure 3). In particular, characteristic shifts in ^1^H NMR spectral peaks compared to the non‐interlocked axle (**A1**‐**A3**) and macrocycle (**M**) components supported the formation of the target interlocked structures. For example, the downfield shift of the triazole resonance (H*_e_*) in rotaxane **CR1** compared to the non‐interlocked axle **A1** (Δδ = 2.31 ppm; Figure 3b,c) was indicative of C‐H···N hydrogen bonding between the triazole and bipyridyl unit of the macrocycle, as previously observed in related systems.^[^ ^44^ ^]^ Cage[*n*]rotaxanes **CR2** and **CR3** (Figure 3d, 3e, respectively), displayed similar resonance shifts compared to their non‐interlocked components. Disappointingly, despite multiple attempts, we were not able to grow X‐ray quality single crystals of any of the cages to confirm their structure in the solid state.

With the target cage[*n*]rotaxanes in hand, we looked to investigate how the properties of the interlocked structures differed from their non‐interlocked counterparts. In this regard we investigated the thermal properties, CO_2_ uptake, and solubilities of the cages, axles, and rotaxanes.

The thermal properties of the cage[*n*]rotaxanes (Table 1) were studied using thermogravimetric analysis (TGA) and optical differential scanning calorimetry (DSC) after activating the samples to remove solvent. The decomposition temperatures (*T*_d_) for non‐interlocked **A1**‐**A3** increased with the number of triazole units from 170 °C (**A1**) to 220 °C (**A3**). In comparison, the rotaxanes **CR1**‐**CR3** all showed lower *T*_d_ values, with limited variation between the different systems (150–160 °C). In contrast to the non‐interlocked axles, which all decomposed prior to melting (Fig. S100, S113, S126), each of the rotaxanes was observed to partially melt (Fig. S141, S155, S169), with this process occurring over a broad temperature range of 10–20 °C, a feature previously observed with other reduced organic cages.^[^ ^49^ ^]^ The apparent degree and onset of melting increased with the number of interlocked components, suggesting the incorporation of the macrocycle, which itself melts at ∼100 °C with a sharp endotherm (Fig. S26, S27), is leading to this observed behavior.

**Table 1:** Decomposition (*T*_d_) and melting temperatures (*T*_m_) for non‐interlocked axles **A1**‐**A3** and rotaxanes **CR1**‐**CR3**.

	*T*_d_ [°C]	*T*_m_ [°C]
**A1**	170	‐
**A2**	180	‐
**A3**	220	‐
**CR1**	150	120–140
**CR2**	160	130–150
**CR3**	160	140–150

**Figure 4:**
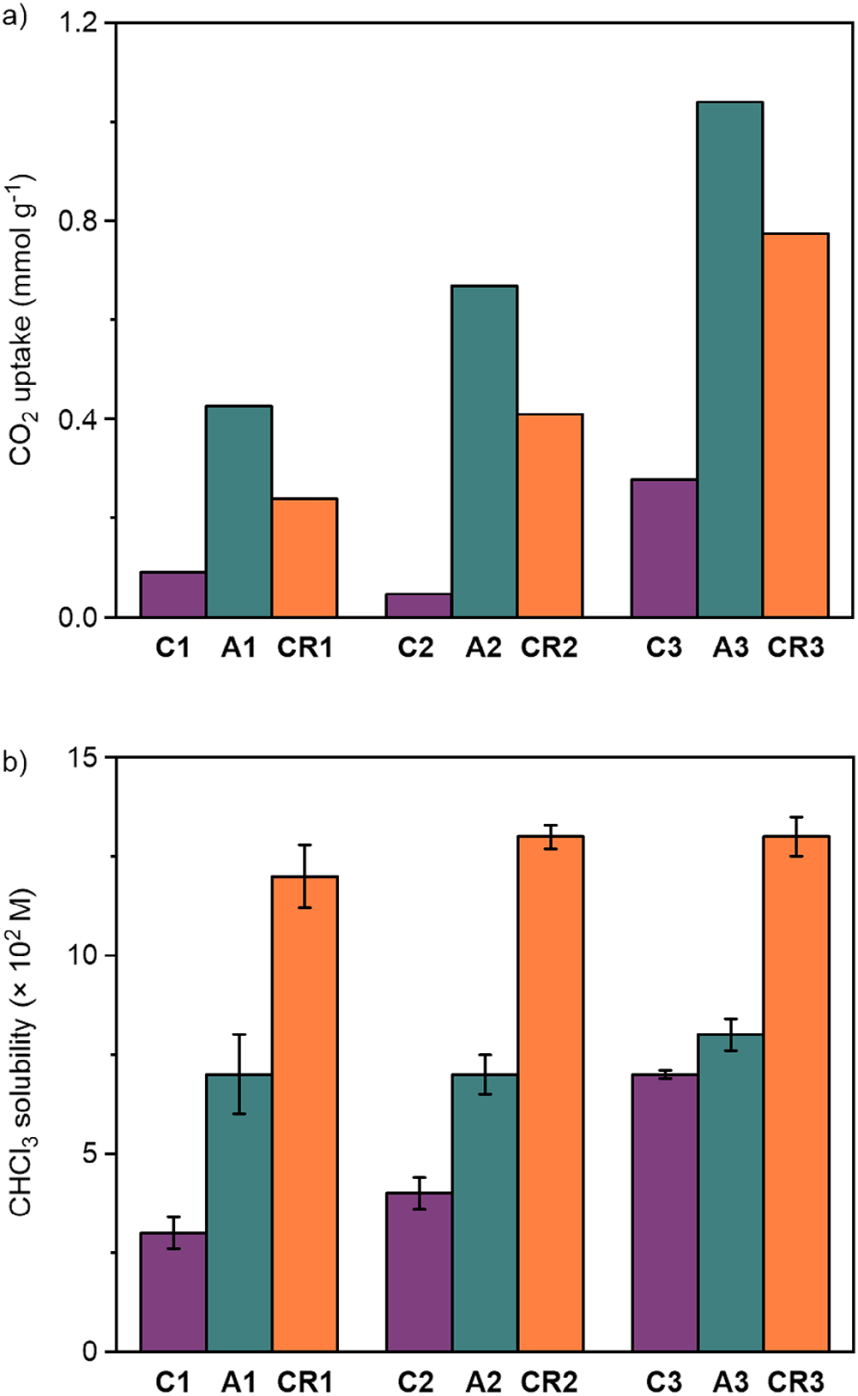
Comparison of the a) CO_2_ uptake (298 K, 1 bar) and b) solubility (CHCl_3_) of non‐interlocked and interlocked cages.

We subsequently shifted our focus to carrying out gas sorption studies (Figure 4a), comparing the alkyne‐functionalized cages (**C1**‐**C3**), non‐interlocked axles (**A1**‐**A3**), and rotaxanes (**CR1**‐**CR3**). Due to the difficulties in purifying large quantities of the cage[*n*]rotaxanes, we performed these on the bulk amorphous samples which, after removing residual azide and macrocycle from the crude material, contained quantities of non‐interlocked axle and/or partially rotaxanated products (14, 7, and 31 mol% for **CR1**, **CR2**, and **CR3**, respectively; see Figure 2). While the rotaxanes all exhibited no porosity to N_2_ (77 K, 1 bar) based on their Brunauer‐Emmett‐Teller (BET) surface areas (4.9, 12.8, and 1.7 m^2^ g^−1^ for **CR1**, **CR2**, and **CR3**, respectively), due to the presence of the free amines in the cage structures, we turned our attention to CO_2_ uptake under standard conditions (298 K, 1 bar). Interestingly, **C3** displayed much higher CO_2_ uptake (0.35 mmol g^−1^) compared to **C1** and **C2** (0.12 and 0.06 mmol g^−1^, respectively). **A1**‐**A3** showed enhanced uptake over their respective alkyne precursors and increased adsorption values with each additional triazole unit (0.42, 0.53, and 0.68 mmol g^−1^ for **A1**, **A2,** and **A3**, respectively). While **C1‐C3** and **A1**‐**A3** might be expected to have chemically identical cavities as the core scaffold on the interior of the cage is identical, they are small and flexible capsules and therefore have limited intrinsic porosity (i.e., porosity arising from the internal voids within a molecule). While we refer to them as a cavity, the largest sphere you could fit into them is 2.1 Å diameter (see Table S5), which is unlikely to be of a sufficient size to host guests. Therefore, we hypothesize the observed trends in CO_2_ uptake are likely to be due to increased extrinsic porosity resulting from poor packing efficiency of the bulky di‐*tert*‐butylphenyl stopper units. Inefficient packing of these systems is unsurprising given their conformational flexibility and anisotropic molecular geometries. Finally, the rotaxanes **CR1**‐**CR3** also showed enhanced uptake with an increasing number of substituents (0.17, 0.19, and 0.26 mmol g^−1^, respectively); however, these values were consistently lower than their non‐interlocked counterparts. While the calculated pore diameters are further decreased for the rotaxanes **CR1**‐**CR3** compared to the corresponding cage axles **A1**‐**A3** (Table S5), we surmise that this decrease is likely more a result of the macrocyclic components occupying the void spaces created by the bulky stopper units, reducing the available extrinsic porosity. In addition, the simulated structures indicate that the macrocyclic components could limit the free space surrounding the amine groups in the cage scaffold. This would additionally frustrate the coordination of CO_2_ molecules with the amine sites, reducing the CO_2_ sorption capacity of the materials containing more macrocyclic components.

As has been previously demonstrated, inter‐component interactions can result in MIMs possessing drastically different solubilities compared to their individual non‐interlocked components.^[^ ^29^, ^30^ ^]^ Consequently, we sought to investigate how the mechanically appended bipyridyl macrocycles affected the solubilities of the cages (Figure 4b), as this is an important factor when processing these species into mixed‐matrix membranes and porous liquids. The non‐interlocked cage axles (**A1**‐**A3**) all displayed similar solubilities (∼0.08 M) in chloroform that were greater than their precursor alkyne cages (**C1**‐**C2**). A further notable enhancement was observed for the rotaxane structures (**CR1**‐**CR3**), with each displaying solubilities of ∼0.13 M. Thus, the presence of **M** (itself displaying high solubility of 0.82±0.032 M) significantly enhanced the solubility of the rotaxanes compared to the non‐interlocked cages. The increase in solubility can be attributed to the increased electropositivity of **CR1**‐**CR3** based on their electrostatic potentials (ESPs, Fig. S210) when compared to the non‐interlocked axles **A1** to **A3**, which contributes to increased solubility in chloroform, a molecule with a slight dipole moment. **CR1**‐**CR3** also has increased ambipolarity, especially within macrocycle‐axle regions, which can also contribute toward chloroform solubility.

## 3 Conclusion

In summary, using a component scrambling methodology followed by reduction, we were able to synthesize robust, amine‐based organic cages with a defined number of alkyne units on the periphery. These functional handles were able to undergo CuAAC reactions, including AMT, to install mechanically interlocked macrocycle components in the formation of [2]‐, [3]‐, and [4]‐rotaxanes. The presence of the macrocycle components was shown to increase the solubility in CHCl_3_ of the cage rotaxanes compared to their non‐interlocked congeners, as well as modify the thermal properties, with rotaxanation inducing partial melting and gas uptake capability of these materials. Modification of these properties would be particularly beneficial in enabling POCs to be studied in different phases, such as neat porous liquids or melt‐quenched glasses, or processed into solution‐based porous liquids. Therefore, this approach offers up an alternative synthetic strategy compared to those previously employed in the area of POC‐based porous liquids, which mainly focus on the incorporation of ionic liquid functionality,^[^ ^50^ ^]^ or scrambling.^[^ ^51^ ^]^ More generally, the ability to post‐synthetically modify POCs using facile CuAAC chemistry opens the door to readily attaching a range of functional units to the cage scaffolds and preparing functionalized POCs from a common precursor. Ultimately, this will allow the precision engineering of (multi‐)functional POCs with customizable properties and behaviors. Studies toward this goal are ongoing in our laboratory.

## Experimental Section

### General synthetic and analytical methods

See Supporting Information for further details.

### Scrambled Synthesis of TIPS‐Alkyne Cages

5‐((Triisopropylsilyl)ethynyl)isophthalaldehyde (**2b**, 1.57 g, 5.0 mmol, 1.0 eq.), isophthalaldehyde (**2a**, 1.34 g, 10.0 mmol, 2.0 eq.), and (2,4,6‐trimethylbenzene‐1,3,5‐triyl)trimethanamine) (**1**, 3.10 g, 14.9 mmol, 3.0 eq.) were dissolved in a mixture of CH_2_Cl_2_/MeOH (1:1, v/v, 600 mL) and stirred at 40 °C for 1 day. The reaction was cooled to room temperature, and sodium borohydride (3.78 g, 100 mmol, 20 eq.) was added in two portions over 2 hours at 0 °C, the second after 2 hours. The mixture was stirred overnight and allowed to warm to room temperature before being quenched with H_2_O (50 mL) and the solvent removed *in vacuo*. A saturated aqueous solution of NaHCO_3_ (100 mL) was added, and the mixture was extracted with CH_2_Cl_2_ (3 × 100 mL). The organic layers were combined, dried (MgSO_4_), filtered, and concentrated *in vacuo* to afford the crude product. The crude product was purified by column chromatography (SiO_2_ (deactivated with NEt_3_) over two runs: the first using isocratic 10% MeOH in *n*hexane:EtOAc (1:1)) to elute the cage species, which was then further purified using a gradient 10% MeOH in *n*hexane:EtOAc (9:1 to 4:1)) to yield the individual cage products.

### Representative procedure for the deprotection of the TIPS‐Alkyne Cages–Mono‐Alkyne Cage (C1)

To a solution of **C1^TIPS^** (1.34 g, 1.50 mmol, 1.0 eq.) in anhydrous THF (30 mL), was added a solution of 1.0 M tetrabutylammonium fluoride in THF (3 mL, 3 mmol, 2 eq.) and the mixture was stirred at room temperature for 2 hours under N_2_. The reaction mixture was quenched with a saturated aqueous NH_4_Cl solution (50 mL), basified to pH 10 with aqueous 1 M NaOH solution, and extracted with Et_2_O (2×100 mL). The organic phases were combined, dried (MgSO_4_), filtered, and concentrated *in vacuo*. EtOH (20 mL) was added to the crude material, and the mixture was sonicated for 10 minutes. The resulting precipitate was collected by filtration to yield the product as a beige solid (717 mg, 0.96 mmol, 64%). **IR** (ν_max_ /cm^−1^) 3283, 2912, 2851, 1595, 1441, 1315, 1116, 902, 725; **^1^H NMR** (400 MHz, CDCl_3_) δ 7.39 (br. app. s, 3H, **H_c,i_**), 7.24—7.21 (m, 4H, **H_b,k_**), 7.10 (d, *J* = 9.3 Hz, 4H, **H_j_**), 3.98 (s, 8H, **H_h_**), 3.93 (s, 4H, **H_d_**), 3.87 (s, 12H, **H_e,g_**), 3.04 (s, 1H, **H_a_**), 2.42 (s, 18H, **H_f_**); **^13^C NMR** (CDCl_3_, 125 MHz) δ_C_: 141.3, 141.0, 136.1, 136.0, 135.1, 134.9, 129.6, 127.8, 125.9, 124.4, 123.5, 121.3, 83.9, 76.8, 54.7, 54.3, 49.6, 15.8; **HRMS** (ESI+) calc. for C_50_H_60_N_6_ [M+H]^+^ 745.4958; found [M+H]^+^ 745.4948.

### Representative procedure for the cage[*n*]rotaxane synthesis–Cage[*2*]rotaxane (CR1)

(*Note*: Stock solutions (5 mg/mL) of tetrakis(acetonitrile)copper(I) hexafluorophosphate ([Cu(MeCN)_4_]PF_6_) (50 mg) in CH_2_Cl_2_ (10 mL) were made before dispensing the correct amount into the reaction mixture, which will be referred to as **solution 1**). To a vial containing (4,7,12,15‐tetraoxa‐1,2(2,6)‐dipyridina‐6,13(1,4)‐dibenzenacyclohexadecaphane) (**M**, 68 mg, 0.14 mmol, 1.0 eq.), **C1** (104 mg, 0.14 mmol, 1 eq.), 3,5‐di‐(*tert*‐butyl)benzyl azide (**S**, 34 mg, 0.14 mmol, 1 eq.), and *N*,*N*‐diisopropylethylamine (44 µL, 0.27 mmol, 2 eq.), under N_2_ in EtOH:CH_2_Cl_2_ (1:1 v/v, 12 mL), was added **solution 1** (1 mL, [Cu(MeCN)_4_]PF_6_ (0.014 mmol, 0.1 eq.)), and the mixture stirred at room temperature for 3 days. The solvent was removed *in vacuo* and to the residue, CH_2_Cl_2_ (10 mL) was added, and the mixture washed with EDTA‐NH_3_ solution (80 mL). The aqueous phase was extracted with CH_2_Cl_2_ (3×50 mL). The organic phases were collected, dried (MgSO_4_), filtered and concentrated *in vacuo*. The crude material was purified by column chromatography, first ((SiO_2_ deactivated with NEt_3_) CH_2_Cl_2_:EtOAc (1:1)) to elute residual azide and macrocycle, and then ((SiO_2_ deactivated with NEt_3_) 10% MeOH in CH_2_Cl_2_:EtOAc (1:1)) to elute the rotaxane species. The crude rotaxane species was purified by reverse phase TLC (RPTLC, C8 SiO_2_ deactivated with NEt_3_, 2% NEt_3_ in MeCN, 6 runs) to yield the product as a colourless solid (20 mg, 0.014 mmol, 10%). **IR** (ν_max_ / cm^−1^) 2948, 2922, 2855, 2358, 2339, 1728, 1608, 1510, 1454, 1249, 1098, 785; **^1^H NMR** (400 MHz, CDCl_3_) δ_H_ 9.67 (s, 1H, **H_e_**), 7.77 (t, J = 7.7 Hz, 2H, **H_H_**), 7.71 (d, J = 7.5 Hz, 2H, **H_G_**), 7.43 (d, J = 7.7 Hz, 2H, **H_I_**), 7.36 (s, 2H, **H_f_**), 7.32 (s, 1H, **H_b_**), 7.29 (s, 1H, **H_g_**), 7.19 (t, J = 7.5 Hz, 2H, **H_m_**), 7.08‐7.00 (m, 8H, **H_c,l,n_**), 6.56–6.45 (m, 8H, **H_C,D_**), 4.83 (s, 2H, **H_d_**), 4.72 (d, J = 8.3 Hz, 2H, two of **H_B_**), 4.42 (d, J = 6.1 Hz, 2H, two of **H_B_**), 4.28 (q, J = 12.1 Hz, 4H, **H_F_**), 3.95 (s, 8H, **H_k_**), 3.85 (s, 8H, **H_j_**), 3.81–3.66 (m, 8H, **H_E_** and **H_h_**), 3.61 (s, 4H, **H_i_**), 2.40 (s, 6H, **H_o_**), 2.38 (s, 12H, **H_p_**), 2.16 (m, 4H, **H_A_**), 1.15 (s, 18H, **H_a_**); **Diffusion coefficient** (500 MHz, CDCl_3_) *D*: 9.46 × 10^−10^ m^2^s^−1^; **^13^C NMR** (101 MHz, CDCl_3_) δ_C_; 159.8, 159.1, 155.4, 151.2, 146.1, 141.2, 139.8, 137.3, 136.0, 135.9, 135.2, 135.0, 134.3, 130.4, 129.3, 127.7, 125.9, 124.3, 123.9, 123.6, 123.3, 121.4, 121.0, 119.9, 115.1, 73.0, 70.2, 66.4, 54.7, 54.1, 49.6, 46.3, 34.8, 31.5, 29.9, 25.0, 15.8; **HRMS** (ESI+) calc. for C_95_H_113_N_11_O_4_ [M+H]^+^ 1472.9055; found [M+H]^+^ 1472.9128.

## Supporting Information

The authors have cited additional references within the Supporting Information.^[^ ^52^, ^53^, ^54^, ^55^, ^56^, ^57^, ^58^, ^59^, ^60^, ^61^ ^]^

## Conflict of Interests

The authors declare no conflicts of interest.

## Supporting information



Supporting information

## Data Availability

The data that support the findings of this study are available in the supplementary material of this article.
