# Supplementary material for: Organic Cage Rotaxanes
Source: Chemistry. 2025 May 27;31(35):e202501014. doi: 10.1002/chem.202501014 (PMC12188171; doi:10.1002/chem.202501014)
Supplement: Supplementary file 1 — Supporting information [file CHEM-31-e202501014-s001.docx]

Supporting Information

Organic Cage Rotaxanes

Zarik Zaheer Yusaf,^[a]^ Benjamin D. Egleston,^[a]^ Gokay Avci,^[a]^ Kim E. Jelfs,^[a]^ James E. M. Lewis^[b]^* and Rebecca L. Greenaway^[a]*^

[a] Z. Z. Yusaf, Dr. B. D. Egleston, Dr. G. Avci, Prof. Dr. K. E. Jelfs, Dr. R. L. Greenaway
Department of Chemistry
Imperial College London
Molecular Sciences Research Hub, White City Campus, Wood Lane, London W12 0BZ, UK.
E-mail: r.greenaway@imperial.ac.uk

[b] Assoc. Prof. Dr. J. E. M. Lewis

School of Chemistry
University of Birmingham
Molecular Sciences Building, Edgbaston, Birmingham B15 2TT, UK.
E-mail: j.e.m.lewis@bham.ac.uk

Table of Contents

[S1: General Experimental	3](#_Toc192598085)

[S2: Synthesis and Characterisation Data	6](#_Toc192598086)

[S2.1. Synthesis of Precursors	6](#_Toc192598087)

[S2.2. Synthesis of Cages	29](#_Toc192598088)

[S2.3. Synthesis of Non-Interlocked Axles	65](#_Toc192598089)

[S2.4. Synthesis of Cage[*n*]Rotaxanes	86](#_Toc192598090)

[S2.5. Synthesis of Cage[*n*]Rotaxane Bulk Species	115](#_Toc192598091)

[S3: Solubility Measurements	136](#_Toc192598092)

[S4: Computational Details	137](#_Toc192598093)

[S5: References	138](#_Toc192598094)

# S1: General Experimental

**Materials:** Chemicals were purchased from TCI UK, Fluorochem, Sigma Aldrich, Acros and Fisher Scientific, and used as received. Solvents were reagent or HPLC grade purchased from VWR except for chloroform-D (CDCl_3_) which was purchased from Fluorochem. All chemicals and solvents were used as received, unless specified. EDTA-NH_3_ solution refers to a 0.1 M solution of EDTA-Na_2_ in 3% NH_3_ (aq).

**Synthesis:** All reactions requiring anhydrous or inert conditions were performed in oven-dried apparatus under an inert atmosphere of dry nitrogen, using anhydrous solvents introduced into the flask using disposable needles and syringes. All reactions were stirred magnetically using Teflon-coated stirring bars. Where heating was required, the reactions were warmed using a stirrer hotplate with heating blocks and the stated temperature was measured externally to the reaction flask with a probe. Removal of solvents was done by rotary evaporation.

**NMR spectra:** ^1^H Nuclear magnetic resonance (NMR) spectra were recorded using an internal deuterium lock for the residual protons in CDCl_3_ (δ = 7.26 ppm) at ambient probe temperature using a Bruker Avance 400 (400 MHz) or Bruker Avance 500 (500 MHz) instrument. NMR data are presented as follows: chemical shift, peak multiplicity (s = singlet, d = doublet, t = triplet, q = quartet, m = multiplet, br = broad, app = apparent), integration, coupling constants (*J* / Hz) and proton assignment. Chemical shifts are expressed in ppm on a δ scale relative to the respective deuterated solvents and coupling constants, *J*, are given in Hz. Assignments were determined either based on unambiguous chemical shift or by analysis of 2D NMR (COSY, HSQC, HMBC). ^13^C NMR spectra were recorded using an internal deuterium lock using solvents CDCl_3_ (δ = 77.16 ppm) at ambient probe temperatures using a Bruker Avance 400 (101 MHz) spectrometer or Bruker Avance 500 (126 MHz) spectrometer.

**Diffusion NMR:** ^1^H DOSY experiments were conducted at 298 K using a Bruker Avance 500 (500MHz) instrument. Processing of DOSY data was achieved using the Bruker Dynamics Centre software version 2.8.4, with an error estimation by Monte Carlo simulation applied at a 95% confidence level. Diffusion data was collected using the Bruker longitudinal eddy current delay (LED) bipolar gradient pulse sequence (ledbpgp2s). A diffusion encoding pulse (δ) of length 2.1 ms and a diffusion delay (Δ) 0.05 s was used. Gradient amplitudes were equally spaced between 0.963 G/cm and 42.757 G/cm. Diffusion coefficients were calculated from signal intensities using the Skejskal-Tanner equation:

$$I=I_{0}e^{-\gamma^{2}G^{2}\delta^{2}\left( \Delta-\frac{\delta}{3} \right)D}$$

Where *I* is the signal intensity, *I_0_* is the signal intensity at a gradient strength of zero, *G* is the gradient strength, and *D* is the diffusion coefficient, γ is the gyromagnetic ratio, δ is the gradient pulse length and Δ is the diffusion time.

**IR spectra:** Infra-red (IR) spectra were obtained on an Agilent Cary 630 FT-IR spectrometer using ATR measurements for oils and solids as neat samples and analysed using Agilent Microloan FTIR software.

**HRMS:** Electrospray ionization mass spectrometry (ES-MS) was carried out using a Waters LCT Premier (ES-ToF) mass spectrometer or an Agilent Technologies 6530B accurate-mass QTOF Dual ESI mass spectrometer (MeOH + 0.1% formic acid, 0.25 mL/min, capillary voltage 4000 V, fragmentor 225 V) in positive-ion detection mode. Values are calculated to 4 decimal places from the molecular formula. The parent ions [M+H]^+^, [M+2H]^2+^, [M+3H]^3+^ are quoted.

**TLC and Chromatography:** Reactions monitored by thin-layer chromatography (TLC) were conducted on pre-coated aluminium-backed plates (Merck Silicagel 60 with fluorescent indicator UV_254_). Spots were visualized under a UV lamp at 254 nm. Where SiO_2_ deactivated with NEt_3_ was used, this refers to the deactivation of silica with 1-3% NEt_3_ in the appropriate mobile phase, followed by running the column with the normal solvent system. Flash column chromatography was conducted on a Teledyne CombiFlash NextGen 100 system. Reverse Preparative TLC (RPTLC) was conducted on pre-coated glass-backed plates (Supelco Silicagel 60 RP-8 with fluorescent indicator UV_254_). Bands were visualised under a UV lamp at 254 nm.

**TGA:** Thermogravimetric analysis was carried out using a Waters TGA550 instrument. The samples were heated in platinum pans under N_2_ at a rate of 10 ⁰C/min to 600 °C.

**PXRD:** Laboratory powder X-ray diffraction data were collected in transmission mode on samples held on a Si low background cavity holder on a Bruker D2 Phaser instrument. Data were measured over the range of 5-40° in ~0.02° steps over 15 minutes.

**Figure S1** - PXRD trace of the blank sample holder used.

**DSC:** Differential scanning calorimetry data were collected using a Linkam Scientific Optical DSC450 instrument. The samples were equilibrated at 25 °C before being heated in aluminium pans under N_2_ at a rate of 10 ⁰C/min to a pre-determined temperature, before being cooled to 25 °C at a rate of 10 ⁰C/min. Samples were prepared in air in Tzero aluminium hermetic pans and left unsealed.

**Figure S2** - DSC trace of blank pan up to 200 °C. DSC traces: Upscan shown as a solid red line and downscan as a blue line.

**Optical Microscopy:** Optical images were taken using the Linkam Scientific LINK Image Capture Module and High Performance Digital Colour Camera (2048x1536), connected to a microscope lens for the Raspberry Pi High Quality Camera - 0.12-1.8x with CS/C mount on the Pimoroni microscope stand. An LED Ring light (76 mm diameter) with a USB to 2.1 mm DC Booster Cable (9V) from The Pi Hut was used around the microscope lens for illumination.

**Gas Sorption Analysis:** Gas uptakes were measured on an Anton Paar Nova 800 system and surface areas were measured by nitrogen sorption at 77 K. Samples were loaded into a 9 mm sample cell. The sample was degassed under vacuum overnight at 90 °C and backfilled with helium before being removed from the degassing station. A filler rod was used with the sample cell during sorption measurements. Gas uptake measurements for CO_2_ (N5.0) were taken at a temperature of 298 K, and N_2_ (N5.5) were taken at a temperature of 77 K.

# S2: Synthesis and Characterisation Data

## S2.1. Synthesis of Precursors

5-Bromoisophthalaldehyde, **S1**

To a multi-necked round bottomed flask, isophthalaldehyde (50.0 g, 373 mmol, 1.0 eq.) was added followed by concentrated sulphuric acid (205 mL, 3.82 mol, 10 eq.) and the resulting mixture was heated to 65 °C. It was then taken off direct heating for the addition of *N*-bromosuccinimide (80.0 g, 449 mmol, 1.2 eq.) over a period of 20 min. After complete addition the heating was resumed, and the reaction was stirred at 65 °C overnight. The reaction mixture was cooled to RT and poured over ice (1 L) and left to settle for 1 h, affording a beige precipitate which was collected by filtration, re-dissolved in _CH2CL2_ (1 L), and washed with H_2_O (2 x 200 mL). The organic phases were collected and dried (MgSO_4_), filtered, and hexane (500 mL) was added. The _CH2CL2_ was carefully removed *in vacuo* and the resulting beige precipitate was collected by filtration and washed with a 1:2 MeOH:Hexane mixture (300 mL) to afford the product which was dried *in vacuo* and used without further purification (37.7 g, 177 mmol, 47%). **^1^H NMR** (CDCl_3_, 400 MHz) δ_H_: 10.06 (s, 2H, **H_c_**), 8.30 (s, 1H, **H_a_**), 8.26 (s, 2H, **H_b_**); **^13^C NMR** (CDCl_3_, 101 MHz) δ_C_: 189.6, 138.6, 137.3, 129.4, 124.5. Data in accordance with the literature.^1^

**Figure S3** - ^1^H NMR (CDCl_3_) spectrum of **S1**.

**Figure S4** - ^13^C NMR (CDCl_3_) spectrum of **S1**.

5-((Triisopropylsilyl)ethynyl)isophthalaldehyde, **2b**

To an oven-dried flask under N_2_ containing **S1** (5.00 g, 23.5 mmol, 1.0 eq.), CuI (135 mg, 0.705 mmol, 0.03 eq.) and bis(triphenylphosphine)palladium(II) dichloride (496 mg, 0.705 mmol, 0.03 eq.) was added anhydrous THF (100 mL) and triethylamine (35 mL) and the mixture was stirred at RT for 5 min. Triisopropylsilylacetylene (9.50 mL, 42.3 mmol, 1.8 eq.) was added dropwise and the reaction was stirred at RT overnight. The crude material was filtered to remove the ammonium salt, and the filtrate was concentrated *in vacuo*. H_2_O (150 mL) was added to the residual material and extracted with CH_2_Cl_2_ (3 x 100 mL). The crude material was purified by flash chromatography (SiO_2_, Hexane:EtOAc (4:1)) to yield the product as a beige solid (6.61 g, 21.0 mmol, 89%). **IR** (ν_max_ /cm^-1^) 3063, 2935, 2860, 2750, 2163, 1705, 1619, 1592, 1454, 698 ; **^1^H NMR** (CDCl_3_, 400 MHz) δ_H_: 10.06 (s, 2H, **H_c_**), 8.27(s, 1H, **H_a_**), 8.17 (s, 2H, **H_b_**), 1.11 (s, 21H, **H_d,e_**); **^13^C NMR** (CDCl_3_, 101 MHz) δ_C_: 190.4, 137.7, 137.1, 129.8, 126.0, 103.9, 95.1, 18.7, 11.3; **HRMS** (ES+) calc. for C_19_H_26_O_2_Si [M+H]^+^ 315.1780; found [M+H]^+^ 315.1775.

**Figure S5** - ^1^H NMR (CDCl_3_) spectrum of **2b.**

**Figure S6** - ^13^C NMR (CDCl_3_) spectrum of **2b.**

**Figure S7** - IR spectrum of **2b.**

**Figure S8** - HRMS spectrum of **2b.**

\#

2,2',2''-((2,4,6-Trimethylbenzene-1,3,5-triyl)tris(methylene))tris(isoindoline-1,3-dione), **S2**

To a solution of 1,3,5-tris(bromomethyl)-2,4,6- trimethylbenzene (20.5 g, 50.1 mmol, 1.0 eq.) and 18-crown-6 (3.97 g, 15.0 mmol, 0.3 eq.) in toluene (450 mL) was added potassium phthalimide (33.4 g, 180.47 mmol, 3.6 eq.). The mixture was heated at 100 °C under N_2_ for 2 days before being allowed to cool to RT. The mixture was concentrated *in vacuo* and the resulting solid suspended in H_2_O (400 mL) and collected by filtration. The resulting solid was further washed with H_2_O (2 × 400 mL) and MeOH (400 mL) before being dried *in vacuo* to afford the desired product as a colourless solid (29.5 g, 49.4 mmol, 99%) which was used without further purification. **^1^H NMR** (400 MHz, CDCl_3_) δ_H_: 7.78 (dd, J = 5.5, 3.1 Hz, 6H, **H_c_**), 7.67 (dd, J = 5.5, 3.1 Hz, 6H, **H_d_**), 4.95 (s, 6H, **H_b_**), 2.50 (s, 9H, **H_a_**); **^13^C NMR** (101 MHz, CDCl_3_) δ_C_: 168.3, 138.7, 134.0, 132.1, 130.3, 123.4, 38.7, 17.5. Data in accordance with the literature.^1^

**Figure S9** - ^1^H NMR (CDCl_3_) spectrum of **S2**.

**Figure S10** - ^13^C NMR (CDCl_3_) spectrum of **S2**.

(2,4,6-Trimethylbenzene-1,3,5-triyl)trimethanamine), **1**

To a suspension of **S2** (7.81 g, 12.9 mmol, 1.0 eq.) in a mixture of toluene (120 mL) and EtOH (300 mL) was added 50-60 wt% aqueous hydrazine hydrate (9.20 mL, 77.4 mmol, 6 eq.) in a single portion. The resulting mixture was heated at 90 °C for 5 days before being allowed to cool to RT and the reaction mixture was concentrated *in vacuo* (not to dryness). The remaining mixture was partitioned between aqueous KOH solution (200 mL, 40 wt%) and CHCl_3_ (300 mL). The aqueous layer was further extracted with CHCl_3_ (2 x 150 mL) before the organic layers were combined, dried (Na_2_SO_4_), filtered and concentrated *in vacuo* to yield the product as a pale-yellow solid (2.44 g, 11.8 mmol, 91%). **^1^H NMR** (400 MHz, CDCl_3_) δ_H_: 3.92 (s, 6H, **H_b_**), 2.45 (s, 9H, **H_a_**); **^13^C NMR** (101 MHz, CDCl_3_) δ_C_: 138.3, 133.6, 41.0, 15.6. Data in accordance with the literature.^1^

**Figure S11** - ^1^H NMR (CDCl_3_) spectrum of **1**.

**Figure S12** - ^13^C NMR (CDCl_3_) spectrum of **1**.

3,5-Di-(*tert*-butyl)benzyl azide, **S**

To an oven-dried flask containing a solution of 3,5-di-(*tert*-butyl)benzyl bromide (1.40 g, 5.0 mmol, 1 eq.) in anhydrous DMF (4.5 mL) was added sodium azide (989 mg, 15.1 mmol, 3 eq.) at RT, and the mixture was stirred overnight under N_2_. H_2_O (150 mL) was added, and the organic material was extracted with Et_2_O (2 x 50 mL), washed with H_2_O (4 x 50 mL), dried (MgSO_4_) and concentrated *in vacuo* to afford the product as a colourless oil (1.11 g, 4.5 mmol, 93%). **^1^H NMR** (400 MHz, CDCl_3_) δ_H_: 7.43 (s, 1H, **H_a_**), 7.17 (s, 2H, **H_b_**), 4.36 (s, 2H, **H_c_**), 1.37 (s, 18H, **H_d_**); **^13^C NMR** (101 MHz, CDCl_3_) δ_C_: 151.5, 134.7, 122.8, 122.5, 55.7, 35.0, 31.6. Data in accordance with the literature.^2^

**Figure S13** - ^1^H NMR (CDCl_3_) spectrum of **S.**

**Figure S14** - ^13^C NMR (CDCl_3_) spectrum of **S.**

Ethyl-6-bromopicolinate, **S3**

A stirring solution of 6-bromo-2-picolinic acid (10.1 g, 33.0 mmol, 1.0 eq.) and H_2_SO_4_ (0.4 mL) in EtOH (80 mL) was heated at reflux for 1 h. The cooled reaction was concentrated *in vacuo* and dissolved in CHCl_3_ (40 mL), washed with 1M aqueous NaOH solution (40 mL) and dried (MgSO_4_), before being concentrated *in vacuo* to yield the product as a colourless solid (9.15 g, 40 mmol, 80%). **^1^H NMR** (CDCl_3_, 400 MHz) δ_H_: 8.08 (d, 1H, J = 8.4 Hz, **H_a_**), 7.70-7.66 (m, 2H, **H_b_** and **H_c_**), 4.47 (q, 2H, J = 7.1 Hz, **H_d_**), 1.43 (t, 3H, J = 7.1 Hz, **H_e_**); **^13^C NMR** (CDCl_3_, 101 MHz) δ_C_: 164.1, 149.3, 142.3, 139.3, 131.8, 124.1, 62.5, 14.4. Data in accordance with the literature.^3^

**Figure S15** – ^1^H NMR (CDCl_3_) spectrum of **S3**.

**Figure S16** - ^13^C NMR (CDCl_3_) spectrum of **S3**.

2-Bromo-6-hydroxymethylpyridine, **S4**

To a solution of **S3** (8.50 g, 37.0 mmol, 1.0 eq.) in EtOH (62.5 mL) in an ice bath under N­_2_ was added NaBH_4_ (1.70 g, 45.0 mmol, 1.2 eq.) portionwise over 1 h. The solution was stirred overnight before being quenched with H_2_O (62.5 mL) resulting in a pink/brown precipitate forming. The EtOH was removed *in vacuo* and the suspension was extracted with CH_2_Cl_2_ (3 x 45 mL). The organic phases were combined, dried (MgSO_4_), and concentrated *in vacuo* to yield the product as a clear oil which solidified into a colourless solid upon standing (5.85 g, 31.0 mmol, 84%). **^1^H NMR** (CDCl_3_, 400 MHz) δ_H_: 7.55 (t, 1H, J = 7.7 Hz, **H_b_**), 7.39 (dd, J = 7.8, 0.5 Hz, 1H, **H_a_**), 7.28 (dd, J = 7.6, 0.6 Hz, 1H, **H_c_**), 4.68 (s, 2H, **H_d_**), 4.16 (s, 1H, **H_e_**); **^13^C NMR** (CDCl_3_, 101 MHz) δ_C_: 161.5, 141.4, 139.2, 126.7, 119.5, 64.3. Data in accordance with the literature.^3^

**Figure S17** – ^1^H NMR (CDCl_3_) spectrum of **S4**.

**Figure S18** – ^13^C NMR (CDCl_3_) spectrum of **S4**.

1,4-Di(4-(hydroxymethyl)phenoxy)butane, **S5**

A solution of 4-hydroxybenzyl alcohol (3.72 g, 30.0 mmol, 2.0 eq.), 1,4-dibromobutane (3.24 g, 15.0 mmol, 1.0 eq.) and K_2_CO_3_ (8.30 g, 60.0 mmol, 4.0 eq.) in MeCN (145 mL) was stirred at reflux for 4 days which afforded a beige precipitate. The solution was reduced to approximately 50 mL *in vacuo* and H_2_O (200 mL) was added, before the beige precipitate was collected by filtration to yield the crude product which was used in the next step without further purification (6.15 g). **^1^H NMR** (CDCl_3_, 400 MHz) δ_H_: 7.28 (d, 4H, J = 8.6 Hz, **H_d_**), 6.89 (d, 4H, J = 8.6 Hz, **H_c_**), 4.62 (d, 4H, J = 5.9 Hz, **H_e_**), 4.04 (t, 4H, J = 5.5 Hz, **H_b_**), 1.98 (t, 4H, J = 2.7 Hz, **H_a_**). Data in accordance with the literature.^3^

**Figure S19** – ^1^H NMR (CDCl_3_) spectrum of **S5**.

1,4-Di(4-(bromomethyl)phenoxy)butane, **S6**

**S5** (6.15 g, 20.0 mmol, 1.0 eq.) was suspended in CH_2_Cl_2_ (37 mL) and HBr (48% w/w aq., 37 mL) and stirred at RT for 3 h until the solid had dissolved. The solution was then diluted with H_2_O (71 mL) and extracted with CHCl_3_ (3 × 40 mL). The combined organic layers were dried (MgSO_4_), filtered and concentrated *in vacuo* to afford crude 1,4-di(4-(bromomethyl)phenoxy)butane as a pale-yellow wax which was used in the next step without further purification (5.63 g). **^1^H NMR** (CDCl_3_, 400 MHz) δ_H_: 7.31 (d, 4H, J = 8.5 Hz, **H_d_**), 6.85 (d, 4H, J = 8.5 Hz, **H_c_**), 4.50 (s, 4H, **H_e­_**), 4.03 (s, 4H, **H_b_**), 1.97 (s, 4H, **H_a_**). Data in accordance with the literature.^3^

**Figure S20** – ^1^H NMR (CDCl_3_) spectrum of **S6**.

1,4-Bis(4-(((6-bromopyridin-2-yl)methoxy)methyl)phenoxy)butane, **S7**

To a solution of **S4** (6.01 g, 32.0 mmol, 2.5 eq.) in anhydrous THF (82 mL) was added 60% NaH in mineral oil (2.43 g, 61.0 mmol, 4.7 eq.) portionwise over 15 min with stirring at 0 °C. **S6** (5.63 g, 13.0 mmol, 1.0 eq.) was added in one portion and the solution stirred for 2 h at RT before being quenched with H_2_O (82 mL). H_2_O (250 mL) was added to the quenched solution resulting in a yellow precipitate, which was collected by filtration, dissolved in CHCl_3_ (82 mL), and washed with brine (82 mL). The organic phase was collected, dried (MgSO_4_), filtered and concentrated *in vacuo*. The crude material was purified by column chromatography (SiO_2_, 2:11:11 EtOAc:Hexane:CH_2_Cl_2_) to give the product as an off-white solid (3.74 g, 5.8 mmol, 45% (54% overall yield across three steps)). **^1^H NMR** (CDCl_3_, 400 MHz) δ_H_: 7.55 (t, 2H, J = 7.7 Hz, **H_h_**), 7.46 (d, 2H, J = 7.5 Hz, **H_i_**), 7.37 (d, 2H, J = 7.8 Hz, **H_g_**), 7.29 (d, 4H, J = 8.7 Hz, **H_d_**), 6.89 (d, 4H, J = 8.6 Hz, **H_c_**), 4.62 (s, 4H, **H_f_**), 4.57 (s, 4H, **H_e_**), 4.04 (m, 4H, **H_b_**), 1.98 (m, 4H, **H_a_**); **^13^C NMR** (CDCl_3_, 101 MHz) δ_C_: 160.6, 158.9, 141.4, 139.1, 129.8, 129.7, 126.7, 120.1, 114.6, 77.2, 72.9, 72.1, 67.6, 26.1. Data in accordance with literature.^3^

**Figure S21** – ^1^H NMR (CDCl_3_) spectrum of **S7**.

**Figure S22** - ^13^C NMR (CDCl_3_) spectrum of **S7**.

(4,7,12,15-Tetraoxa-1,2(2,6)-dipyridina-6,13(1,4)-dibenzenacyclohexadecaphane), **M**

**Note:** Pre-washing syringes with DMF was carried out before making the catalyst mixture and dispensing the precursor solution via syringe. In addition, the highest stirring rate was used.

Dibromobis(triphenylphosphine)nickel(II) (1.49 g, 2.02 mmol, 1 eq.), triphenylphosphine (1.05 g, 4.00 mmol, 2 eq.), Mn (1.10 g, 20.0 mmol, 10 eq.) and tetraethylammonium iodide (0.514 g, 2.00 mmol, 1 eq.) in anhydrous DMF (15 mL) were sonicated for 10 min, causing a change in colour from green to dark red. The mixture was then stirred at 50 °C for 1 h. To this catalyst mixture was added **S7** (1.28 g, 2.01 mmol, 1 eq.) in anhydrous DMF (15 mL) *via* syringe over 4 h, followed by additional stirring for 1 h. To the cooled reaction was added CH_2_Cl_2_ (100 mL), followed by washing with EDTA-NH_3_ solution (100 mL). The organic phase was collected and washed with water (5 x 200 mL), followed by brine (100 mL). The organic phase was collected, dried (MgSO_4_), filtered and concentrated *in vacuo*. The crude material was purified by flash chromatography (SiO_2_ (deactivated with NEt_3_), Hexane:Et_2_O (0 to 75%)) to yield the product as a colourless solid (0.465 g, 0.96 mmol, 48%). **^1^H NMR** (400 MHz, CDCl_3_) δ_H_ 7.73-7.72 (m, 4H, **H_g,h_**), 7.43 (d, J = 8.8 Hz, 2H, **H_i_**), 7.24 (d, J = 8.6 Hz, 4H, **H_d_**), 6.76 (d, J = 8.7 Hz, 4H, **H_c_**), 4.65 (s, 4H, **H_f_**), 4.54 (s, 4H, **H_e_**), 4.03 (t, J = 5.8 Hz, 4H, **H_b_**), 1.96 (t, J = 5.8 Hz, 4H, **H_a_**); **^13^C NMR** (101 MHz, CDCl_3_) δ_C_ 159.2, 158.8, 156.0, 137.2, 130.7, 129.7, 121.5, 120.6, 114.8, 77.2, 72.8, 72.0, 67.4, 25.4. Data in accordance with the literature.^4^

**Figure S23** – ^1^H NMR (CDCl_3_) spectrum of **M**.

**Figure S24** - ^13^C NMR (CDCl_3_) spectrum of **M**.

**Figure S25** – TGA trace with plotted first derivative of **M**.

**Figure S26** - DSC traces and corresponding TGA curve (black) of **M** subjected to heat-cool-heat-cool (HCHC) cycles to 220 °C. DSC traces: Initial upscan shown as a solid red line, downscan as a blue line, second upscan as a dashed red line and second downscan as a dashed blue line.

**Figure S27** - Optical images of desolvated **M** at different stages subjected to HCHC cycles to 220 °C.

**Figure S28** - ^1^H NMR (CDCl_3_) of **M** post DSC.

**Figure S29** - Stacked PXRDs of **M**; neat macrocycle (black) and post DSC to 220 °C (orange).

## S2.2. Synthesis of Cages

Mono-Alkyne Cage, **C1**

To a solution of **C1^TIPS^** (1.34 g, 1.50 mmol, 1.0 eq.) in anhydrous THF (30 mL), a solution of 1.0 M tetrabutylammonium fluoride in THF (3 mL, 3 mmol, 2 eq.) was added and the mixture was stirred at RT for 2 h under N_2_. The reaction mixture was quenched with saturated NH_4_Cl solution (50 mL), basified to pH 10 with aqueous 1M NaOH solution, and extracted with Et_2_O (2 x 100 mL). The organic phases were combined, dried (MgSO_4_), filtered and concentrated *in vacuo*. EtOH (20 mL) was added to the crude material and sonicated for 10 min. The mixture was filtered, and the precipitate collected by filtration to yield the product as a beige solid (717 mg, 0.96 mmol, 64%). **IR** (ν_max_ /cm^-1^) 3283, 2912, 2851, 1595, 1441, 1315, 1116, 902, 725; ^1^H NMR (400 MHz, CDCl_3_) δ 7.39 (br. app. s, 3H, **H_c,i_**), 7.24 – 7.21 (m, 4H, **H_b,k_**), 7.10 (d, *J* = 9.3 Hz, 4H, **H_j_**), 3.98 (s, 8H, **H_h_**), 3.93 (s, 4H, **H_d_**), 3.87 (s, 12H, **H_e,g_**), 3.04 (s, 1H, **H_a_**), 2.42 (s, 18H, **H_f_**); **^13^C NMR** (CDCl_3_, 125 MHz) δ_C_: 141.3, 141.0, 136.1, 136.0, 135.1, 134.9, 129.6, 127.8, 125.9, 124.4, 123.5, 121.3, 83.9, 76.8, 54.7, 54.3, 49.6, 15.8 ; **HRMS** (ESI+) calc. for C_50_H_60_N_6_ [M+H]^+^ 745.4958; found [M+H]^+^ 745.4948.

**Figure S30** - ^1^H NMR (CDCl_3_) spectrum of **C1**.

**Figure S31** - ^1^H-^1^H COSY NMR (CDCl_3_) spectrum of **C1**.

**Figure S32** - ^13^C NMR (CDCl_3_) spectrum of **C1**.

**Figure S33** - ^1^H-^13^C HSQC NMR (CDCl_3_) spectrum of **C1**.

**Figure S34** - ^1^H-^13^C HMBC NMR (CDCl_3_) spectrum of **C1**.

**Figure S35** - IR spectrum of **C1**.

**Figure S36** - HRMS spectrum of **C1**.

**Figure S37** - TGA trace with plotted first derivative of **C1**.

**Figure S38** - DSC traces and corresponding TGA curve (black) of **C1** subjected to heat-cool-heat-cool (HCHC) cycles to 200 °C. DSC traces: Initial upscan shown as a solid red line, downscan as a blue line, second upscan as a dashed red line and second downscan as a dashed blue line.

**Figure S39** - Optical images of desolvated **C1** at different stages subjected to HCHC cycles to 200 °C.

**Figure S40** - ^1^H NMR (CDCl_3_) of **C1** post DSC.

**Figure S41** – CO_2_ adsorption (filled) and desorption (empty) isotherms for **C1**.

**Figure S42** - Stacked PXRDs of **C1**; solvated (black), desolvated (blue), post-sorption (green) and post DSC to 200 °C (orange).

Di-Alkyne Cage, **C2**

To a solution of **C2^TIPS^** (0.524 g, 0.48 mmol, 1.0 eq.) in anhydrous THF (20 mL), a solution of 1.0 M tetrabutylammonium fluoride in tetrahydrofuran (1.95 mL, 1.95 mmol, 4.1 eq.) was added and the mixture was stirred at RT for 2 h under N_2_. The reaction mixture was quenched with saturated NH_4_Cl solution (50 mL), basified to pH 10 with aqueous 1 M NaOH solution and extracted with Et_2_O (3 x 100 mL). The organic phases were combined, dried (MgSO_4_), filtered, and concentrated *in vacuo*. EtOH (10 mL) was added to the crude material and sonicated for 10 min. The mixture was filtered, and the precipitate collected by filtration to yield the product as a beige solid (0.2689 g, 0.35 mmol, 73%). **IR** (ν_max_ / cm^-1^) 3280, 2852, 1594, 1446, 1319, 1071, 650; **^1^H NMR** (400 MHz, CDCl_3_) δ_H_ 7.39 (br. app. s, 3H, **H_c,i_**), 7.24 – 7.20 (m, 5H, **H_b,k_**), 7.09 (d, *J* = 5.9 Hz, 2H, **H_j_**), 3.97 (s, 4H, **H_h_**), 3.93 (s, 8H, **H_d_**), 3.86 (s, 12H, **H_e,g_**), 3.04 (s, 2H, **H_a_**), 2.41 (s, 18H, **H_f_**); **^13^C NMR** (101 MHz, CDCl_3_) δ_C_: 141.31, 141.08, 136.05, 135.14, 134.95, 129.61, 127.77, 125.92, 124.43, 123.44, 121.35, 83.91, 54.72, 54.29, 49.53, 15.78. **HRMS** (ESI+) calc. for C_52_H_60_N_6_ [M+H]^+^ 769.4958; found [M+H]^+^ 769.4969.

**Figure S43** - ^1^H NMR (CDCl_3_) spectrum of **C2**.

**Figure S44** – ^1^H-^1^H COSY NMR (CDCl_3_) spectrum of **C2**.

**Figure S45** - ^13^C NMR (CDCl_3_) spectrum of **C2**.

**Figure S46** - ^1^H-^13^C HSQC NMR (CDCl_3_) spectrum of **C2**.

**Figure S47** - ^1^H-^13^C HMBC NMR (CDCl_3_) spectrum of **C2**.

**Figure S48** - IR spectrum of **C2**.

**Figure S49** - HRMS spectrum of **C2**.

**Figure S50** - TGA trace and plotted first derivative of **C2**.

**Figure S51** - DSC traces and corresponding TGA curve (black) of **C2** subjected to heat-cool-heat-cool (HCHC) cycles to 200 °C. DSC traces: Initial upscan shown as a solid red line, downscan as a blue line, second upscan as a dashed red line and second downscan as a dashed blue line.

**Figure S52** - Optical images of desolvated **C2** at different stages subjected to HCHC cycles to 200 °C.

**Figure S53** - ^1^H NMR (CDCl_3_) of **C2** post DSC.

**Figure S54** – CO_2_ adsorption (filled) and desorption (empty) isotherms for **C2**.

**Figure S55** - Stacked PXRDs of **C2**; solvated (black), desolvated (blue), post-sorption (green) and post DSC to 200 °C (orange).

Tri-Alkyne Cage, **C3**

To a solution of **C3^TIPS^** (3.80 g, 3.0 mmol, 1.0 eq.) in anhydrous THF (60 mL), a solution of 1.0 M tetrabutylammonium fluoride in THF  (18.0 mL, 18.0 mmol, 6 eq.)  was added and the mixture was stirred at RT for 2 h under N_2_. The reaction mixture was quenched with saturated NH_4_Cl solution (100 mL), basified to pH 10 with aqueous 1 M NaOH solution and extracted with Et_2_O (3 x 150 mL). The organic phases were combined, dried (MgSO_4_), filtered, and concentrated *in vacuo*. MeOH (50 mL) was added to the crude material and sonicated for 10 min. The mixture was filtered, and the precipitate collected to yield the product as an off-white solid (0.990 g, 1.24 mmol, 41%). **IR** (ν_max_ / cm^-1^) 3283, 2915, 2855, 2100, 1593, 1450, 1111, 858, 651, 600; **^1^H NMR** (400 MHz, CDCl_3_) δ_H_: 7.38 (s, 3H, H**_c_**), 7.25 – 7.22 (m, 6H, H**_b_**), 3.92 (s, 12H, H**_d_**), 3.85 (s, 12H, H**_e_**), 3.04 (s, 3H, H**_a_**), 2.40 (s, 18H, H**_f_**); **^13^C NMR** (101 MHz, CDCl_3_) δ_C_: 141.2, 136.0, 134.9, 129.7, 124.6, 121.4, 83.9, 77.2, 54.3, 49.5, 15.8; **HRMS** (ESI+) calc. for C_54_H_60_N_6_ [M+H]^+^ 793.4958; found [M+H]^+^ 793.4954.

**Figure S56** - ^1^H NMR (CDCl_3_) spectrum of **C3**.

**Figure S57** - ^13^C NMR (CDCl_3_) spectrum of **C3**.

**Figure S58** - IR spectrum of **C3**.

**Figure S59** - HRMS spectrum of **C3**.

**Figure S60** - TGA trace with plotted first derivative of **C3**.

**Figure S61** - DSC traces and corresponding TGA curve (black) of **C3** subjected to heat-cool-heat-cool (HCHC) cycles to 200 °C. DSC traces: Initial upscan shown as a solid red line, downscan as a blue line, second upscan as a dashed red line and second downscan as a dashed blue line.

**Figure S62** - Optical images of desolvated **C3** at different stages subjected to HCHC cycles to 200 °C.

**Figure S63** - ^1^H NMR (CDCl_3_) of **C3** post DSC.

**Figure S64** – CO_2_ adsorption (filled) and desorption (empty) isotherms for **C3**.

**Figure S65** - Stacked PXRDs of **C3**; solvated (black), desolvated (blue), post-sorption (green) and post DSC to 200 °C (orange).

Scrambled Synthesis of TIPS-Alkyne Cages

**General procedure**: **2b** (1.57 g, 5.0 mmol, 1.0 eq.), isophthalaldehyde (1.34 g, 10.0 mmol, 2.0 eq.) and **1** (3.10 g, 14.9 mmol, 3.0 eq.) were dissolved in a mixture of CH_2_Cl_2_/MeOH (1:1, v/v, 600 mL) and stirred at 40 °C for 1 day. The reaction was cooled to RT and sodium borohydride (3.78 g, 100 mmol, 20 eq.) was added in two portions over 2 h at 0 °C, the second after 2 h. The mixture was stirred overnight and allowed to warm to RT before being quenched with H_2_O (50 mL) and the solvent removed *in vacuo*. A saturated aqueous solution of NaHCO_3_ (100 mL) was added, and the mixture was extracted with CH_2_Cl_2_ (3 × 100 mL). The organic layers were combined, dried (MgSO_4_), filtered and concentrated *in vacuo* to yield the crude product. The crude product was initially purified by column chromatography (SiO_2_ (deactivated with NEt_3_) 10% MeOH in Hexane:EtOAc (1:1)) to elute the cage species. This was then further purified by column chromatography (SiO_2_ (deactivated with NEt_3_) 10% MeOH in Hexane:EtOAc (9:1 to 4:1)) to yield the individual cage products.

Parent Unsubstituted Cage, **C4**

Following the above general procedure, this was isolated as an off-white solid (0.750 g, 1.04 mmol, 17%). **^1^H NMR** (CDCl_3_, 400 MHz) δ_H_: 7.40 (s, 3H, **H_a_**), 7.25 – 7.20 (m, 3H, **H_c_**), 7.10 (d, *J* = 6.0 Hz, 6H, **H_b_**), 3.98 (s, 12H, **H_d_**), 3.87 (s, 12H, **H_e_**), 2.43 (s, 18H, **H_f_**); **^13^C NMR** (CDCl_3_, 101 MHz) δ_C_: 141.0, 136.0, 135.1, 127.8, 125.9, 123.5, 77.2, 54.7, 49.6, 18.8, 15.8, 11.4; **HRMS** (ESI+) calc. for C_48_H_60_N_6_ [M+H]^+^ 721.4958; found [M+H]^+^ 721.4934. Data in accordance with the literature.

**Figure S66** - ^1^H NMR (CDCl_3_) spectrum of **C4**.

**Figure S67** - ^13^C NMR (CDCl_3_) spectrum of **C4**.

**Figure S68** - HRMS spectrum of **C4**.

Mono-TIPS Alkyne Cage, **C1^TIPS^**

Following the above general procedure, this was isolated as an off-white solid (0.883 g, 0.98 mmol, 20%). **IR** (ν_max_ /cm^-1^) 2919, 2861, 1709, 1596, 1447, 1117, 1069, 881, 731, 670, 497; **^1^H NMR** (400 MHz, CDCl_3_) δ_H_ 7.39 (s, 2H, **H_j_**), 7.36 (s, 1H, **H_d_**), 7.25 – 7.20 (m, 4H, **H_c,k_­**), 7.10 (d, *J* = 7.6 Hz, 4H, **H_l_**), 3.97 (s, 8H, **H_i_**), 3.92 (s, 4H, **H_e_**), 3.87 (s, 12H, **H_e,h_**), 2.42 (s, 18H, **H_g_**), 1.14 (s, 21H, **H_a,b_**); **^13^C NMR** (CDCl_3_, 101 MHz) δ_C_: 141.1, 141.0, 136.0, 136.0, 135.1, 135.0, 129.6, 127.8, 125.9, 123.5, 107.4, 90.0, 77.5, 77.2, 54.7, 54.4, 49.6, 18.8, 15.8, 11.5; **HRMS** (ESI+) calc. for C_59_H_80_N_6_Si [M+H]^+^ 901.6292; found [M+H]^+^ 901.6334.

**Figure S69** - ^1^H NMR (CDCl_3_) spectrum of **C1^TIPS^**.

**Figure S70** - ^1^H-^1^H COSY NMR (CDCl_3_) spectrum of **C1^TIPS^**.

**Figure S71** - ^13^C NMR (CDCl_3_) spectrum of **C1^TIPS^**.

**Figure S72** - ^1^H-^13^C DEPT-edited HSQC NMR (CDCl_3_) spectrum of **C1^TIPS^**.

**Figure S73** - ^1^H-^13^C HMBC NMR (CDCl_3_) spectrum of **C1^TIPS^**.

**Figure S74** - IR spectrum of **C1^TIPS^**.

**Figure S75** - HRMS spectrum of **C1^TIPS^**.
Di-TIPS Alkyne Cage, **C2^TIPS^**

Following the above general procedure, this was isolated as a crystalline colourless solid (0.650 g, 0.60 mmol, 14%). **IR** (ν_max_ /cm^-1^) 2923, 2859, 2147, 1726, 1596, 1448, 1117, 1067, 880, 738, 670; **^1^H NMR** (400 MHz, CDCl_3_) δ_H_ 7.39 (s, 1H, **H_j_**), 7.36 (s, 2H, **H_d_**), 7.24-7.20 (m, 5H, **H_c,k_**), 7.10 (d, *J* = 8.4 Hz, 2H, **H_l_**), 3.97 (s, 4H, **H_i_**), 3.92 (s, 8H, **H_e_**), 3.85 (s, 12H, **H_f,h_**), 2.41 (s, 18H, **H_g_**), 1.14 (s, 42H, **H_a,b_**); **^13^C NMR** (CDCl_3_, 101 MHz) δ_C_: 141.1, 141.1, 136.0, 135.1, 135.0, 129.6, 127.8, 125.9, 124.2, 123.5, 122.8, 107.4, 90.0, 77.2, 54.7, 54.4, 49.5, 18.8, 15.8, 11.5; **HRMS** (ESI+) calc. for C­_70_H_100_N_6_Si_2_ [M+H]^+^ 1081.7626; found [M+H]^+^ 1081.7677.

**Figure S76** - ^1^H NMR (CDCl_3_) spectrum of **C2^TIPS^**.

**Figure S77** - ^1^H-^1^H COSY NMR (CDCl_3_) spectrum of **C2^TIPS^**.

**Figure S78** - ^13^C NMR (CDCl_3_) spectrum of **C2^TIPS^**.

**Figure S79** - ^1^H-^13^C DEPT-edited HSQC NMR (CDCl_3_) spectrum of **C2^TIPS^**.

**Figure S80** - ^1^H-^13^C HMBC NMR (CDCl_3_) spectrum of **C2^TIPS^**.

**Figure S81** - IR spectrum of **C2^TIPS^**.

**Figure S82** - HRMS spectrum of **C2^TIPS^**.

Tri-TIPS Alkyne Cage, **C3^TIPS^**

Following the above general procedure, this was isolated as a slightly yellow powder (0.110 g, 0.09 mmol, 2%). **IR** (ν_max_ /cm^-1^) 2930, 2859, 2157, 1738, 1597, 1454, 1242, 884, 735, 671; **^1^H NMR** (CDCl_3_, 400 MHz) δ_H_: 7.37 (s, 3H, **H_d_**), 7.25 (d, *J* = 1.7 Hz, 6H, **H_c_**), 3.93 (s, 12H, **H_e_**), 3.86 (s, 12H, **H_f_**), 2.42 (s, 18H, **H_g_**), 1.15 (s, 63H, **H_a,b_**); **^13^C NMR** (CDCl_3_, 101 MHz) δ_C_: 141.1, 136.0, 135.0, 129.7, 124.3, 122.9, 107.4, 90.0, 77.2, 54.4, 49.5, 18.8, 15.8, 11.5; **HRMS** (ESI+) calc. for C_81_H_120_N_6_Si_3_ [M+H]^+^ 1261.8961; found [M+H]^+^ 1261.8942.

**Figure S83** - ^1^H NMR (CDCl_3_) spectrum of **C3^TIPS^**.

**Figure S84** - ^13^C NMR (CDCl_3_) spectrum of **C3^TIPS^**.

**Figure S85** - IR spectrum of **C3^TIPS^**.

**Figure S86** - HRMS spectrum of **C3^TIPS^**.

Tri-TIPS-Alkyne Cage, **C3^TIPS^**

**2b** (2.85 g, 9.0 mmol, 3.0 eq.) and **1** (1.86 g, 9.0 mmol, 3.0 eq.) were dissolved in a mixture of CH_2_Cl_2_:MeOH (1:1, v/v, 700 mL) and stirred at 40 °C for 2 days. The reaction mixture was cooled to RT and sodium borohydride (5.79 g, 153 mmol, 51.0 eq.) was added in two batches, the second after 2 h, and the mixture stirred in a water bath overnight. The mixture was quenched with H_2_O (100 mL) and the solvent removed *in vacuo*. To the residue, saturated aqueous NaHCO_3_ solution (200 mL) was added, and the mixture extracted with CH_2_Cl_2_ (3 x 200 mL). The organic layers were collected, dried (MgSO_4_), and concentrated *in vacuo* to give the product as a pale-pink solid (3.8067 g, 3.0 mmol, quant.). **IR** (ν_max_ /cm^-1^) 2935, 2920, 2858, 2790, 2150, 1734, 1595, 1449, 1242, 715 ; **^1^H NMR** (CDCl_3_, 400 MHz) δ_H_: 7.35 (s, 3H, **H_d_**), 7.22 (s, 6H, **H_c_**), 3.91 (s, 12H, **H_e_**), 3.85 (s, 12H, **H_f_**), 2.40 (s, 18H, **H_g_**), 1.13 (s, 63H, **H_a,b_**); **^13^C NMR** (CDCl_3_, 101 MHz) δ_C_: 141.1, 136.0, 135.0, 129.6, 124.3, 122.9, 107.3, 90.0, 54.4, 49.5, 18.8, 15.8, 11.4; **HRMS** (ESI+) calc. for C_81_H_120_N_6_Si_3_ [M+H]^+^ 1261.8961 and [M+2H]^2+^ 631.4519; found [M+H]^+^ 1261.8940, [M+2H]^2+^ 631.4510.

**Figure S87** - ^1^H NMR (CDCl_3_) spectrum of **C3^TIPS^**.

**Figure S88** - ^13^C NMR (CDCl3) spectrum of **C3^TIPS^**.

**Figure S89** - IR spectrum of **C3^TIPS^**.

**Figure S90** - HRMS spectrum and expanded sections of **C3^TIPS^**.

## S2.3. Synthesis of Non-Interlocked Axles

Cage[2]Rotaxane Non-Interlocked Axle, **A1**

**S** (98 mg, 0.4 mmol, 2 eq.), **C1** (149 mg, 0.2 mmol, 1.0 eq.), sodium ascorbate (119 mg, 0.6 mmol, 3.0 eq.) and copper(II) sulfate pentahydrate (75 mg, 0.3 mmol, 1.5 eq.) were stirred in anhydrous DMF (3 mL) at RT for 3 days under N_2_. EDTA-NH_3_ solution (50 mL) was added, and the mixture extracted with CH_2_Cl_2_ (3 x 150 mL). The organic phases were combined and washed with brine (150 mL) collected, dried (MgSO_4_), filtered and concentrated *in vacuo*. The crude material was azeotroped with toluene to remove the bulk of the DMF and then re-dissolved in CH_2_Cl_2_ (100 mL) and hexane (200 mL) added. The _­_CH_2_Cl_2_ was removed *in vacuo* and the resulting precipitate filtered and dried to yield the product as an off-white solid (75 mg, 0.08 mmol, 38%); **^1^H NMR** (400 MHz, CDCl_3_) δ_H_ 7.68 (s, 1H, **H_e_**), 7.56 (s, 2H, **H_f_**), 7.45 (s, 1H, **H_b_**), 7.39 (s, 2H, **H_o_**), 7.36 (s, 1H, **H_g_**), 7.25 – 7.19 (m, 2H, **H_n_**), 7.16 (s, 2H, **H_c_**), 7.10 (d, J = 7.7 Hz, 4H, **H_m_**), 5.56 (s, 2H, **H_d_**), 3.99 (s, 4H, **H_h_**), 3.97 (s, 8H, **H_k_**), 3.87 (s, 12H, **H_i,l_**), 2.42 (s, 18H, **H_j_**), 1.33 (s, 18H, **H_a_**); **^13^C NMR** (126 MHz, CDCl_3_) δ_C_ 152.0, 148.2, 141.7, 141.0, 136.0, 135.1, 134.0, 130.1, 129.3, 127.8, 126.0, 123.6, 123.4, 123.0, 122.5, 119.6, 55.0, 54.7, 54.6, 49.6, 36.6, 35.1, 31.6, 22.8, 15.8; **HRMS** (ESI+) calc. for C_65_H_83_N_9_ [M+H]^+^ 990.6850; found [M+H]^+^ 990.6882.

**Figure S91** - ^1^H NMR (CDCl_3_) spectrum of **A1**.

**Figure S92** - ^1^H-^1^H COSY NMR (CDCl_3_) spectrum of **A1**.

**Figure S93** - ^13^C NMR (CDCl_3_) spectrum of **A1**.

**Figure S94** - ^1^H-^13^C HSQC NMR (CDCl_3_) spectrum of **A1**.

**Figure S95** - ^1^H-^13^C HMBC NMR (CDCl_3_) spectrum of **A1**.

**Figure S96** - IR spectrum of **A1**.

**Figure S97** - HRMS spectrum of **A1**.

**Figure S98** - TGA trace with plotted first derivative of **A1**.

**Figure S99** - DSC traces and corresponding TGA trace (black) of **A1** subjected to heat-cool-heat-cool (HCHC) cycles to 150 °C. DSC traces: Initial upscan shown as a solid red line, downscan as a blue line, second upscan as a dashed red line and second downscan as a dashed blue line.

**Figure S100** - Optical images of desolvated **A1** at different stages subjected to HCHC cycles to 150 °C.

**Figure S101** - ^1^H NMR (CDCl_3_) of **A1** post DSC.

**Figure S102** – CO_2_ adsorption (filled) and desorption (empty) isotherms for **A1**.

**Figure S103** - Stacked PXRDs of **A1**; solvated (black), desolvated (blue), post-sorption (green) and post DSC to 150 °C (orange).

Cage[3]Rotaxane Non-Interlocked Axle, **A2**

**S** (59 mg, 0.24 mmol, 3 eq.), **C2** (62 mg, 0.08 mmol, 1.0 eq.), sodium ascorbate (48 mg, 0.24 mmol, 3.0 eq.) and copper(II) sulfate pentahydrate (30 mg, 0.12 mmol, 1.5 eq.) were stirred in anhydrous DMF (3 mL) at RT for 3 days under N_2_. EDTA-NH_3_ solution (50 mL) was added, and the mixture extracted with CH_2_Cl_2_ (3 x 150 mL). The organic phases were combined and washed with brine (150 mL) collected, dried (MgSO_4_), filtered and concentrated *in vacuo*. The crude material was azeotroped with toluene to remove the bulk of the DMF and then re-dissolved in CH_2_Cl_2_ (100 mL) and hexane (200 mL) added. The CH_2_Cl_2_ was removed *in vacuo* and the resulting precipitate filtered and dried to yield the product as an off-white solid (51 mg, 0.04 mmol, 51%); **IR** (ν_max_ / cm^-1^) 3008, 2963, 2859, 1663, 1600, 1444, 1215, 745; **^1^H NMR** (500 MHz, CDCl_3_) δ_H_ 7.66 (s, 2H, **H_e_**), 7.55 (s, 4H, **H_c_**), 7.44-7.43 (m, 3H, **H_b,o_**), 7.40-7.37 (m, 1H, **H_n_**), 7.35 (s, 2H, **H_g_**), 7.15(s, 4H, **H_f_**), 7.08 (d, *J* = 7.9 Hz, 2H, **H_m_**), 5.55 (s, 4H, **H_d_**), 3.97 (d, *J* = 11.4 Hz, 12H, **H_h,l_**), 3.85 (s, 12H, **H_i,k_**), 2.41 (s, 18H, **H_j_**), 1.31 (s, 36H, **H_a_**); **^13^C NMR** (126 MHz, CDCl_3_) δ_C_ 152.0, 148.2, 141.7, 141.0, 136.0, 135.1, 134.0, 130.1, 127.8, 125.9, 123.7, 123.3, 123.0, 122.5, 119.6, 55.0, 54.6, 49.5, 35.1, 31.6, 15.8; **HRMS** (ESI+) calc. for C_82_H_106_N_12_ [M+H]^+^ 1259.8742; found [M+H]^+^ 1259.8765.

**Figure S104** - ^1^H NMR (CDCl_3_) spectrum of **A2**.

**Figure S105** - ^1^H-^1^H COSY NMR (CDCl_3_) spectrum of **A2**.

**Figure S106** - ^13^C NMR (CDCl_3_) spectrum of **A2**.

**Figure S107** - ^1^H-^13^C HSQC NMR (CDCl_3_) spectrum of **A2**.

**Figure S108** - ^1^H-^13^C HMBC NMR (CDCl_3_) spectrum of **A2**.

**Figure S109** - IR spectrum of **A2**.

**Figure S110** - HRMS spectrum of **A2**.

**Figure S111** - TGA trace with plotted first derivative of **A2**.

**Figure S112** - DSC traces and corresponding TGA curve (black) of **A2** subjected to heat-cool-heat-cool (HCHC) cycles to 150 °C. DSC traces: Initial upscan shown as a solid red line, downscan as a blue line, second upscan as a dashed red line and second downscan as a dashed blue line.

**Figure S113** - Optical images of desolvated **A2** at different stages subjected to HCHC cycles to 150 °C.

**Figure S114** – ^1^H NMR (CDCl_3_) spectrum of **A2** post DSC.

**Figure S115** – CO_2_ adsorption (filled) and desorption (empty) isotherms for **A2**.

**Figure S116** - Stacked PXRDs of **A2**; solvated (black), desolvated (blue), post-sorption (green) and post DSC to 150 °C (orange).

Cage[4]Rotaxane Non-Interlocked Axle, **A3**

**S** (128 mg, 0.52 mmol, 4 eq.), **C3** (103 mg, 0.13 mmol, 1.0 eq.), sodium ascorbate (77 mg, 0.39 mmol, 3.0 eq.) and copper(II) sulfate pentahydrate (50 mg, 0.2 mmol, 1.5 eq.) were stirred in anhydrous DMF (5 mL) at RT for 3 days under N_2_. EDTA-NH_3_ solution (300 mL) was added, and the mixture extracted with CH_2_Cl_2_ (3 x 150 mL). The organic phases were combined and washed with H_2_O (4 x 50 mL), collected, dried (MgSO_4_), filtered and concentrated *in vacuo*. The crude material was azeotroped with toluene to remove the bulk of the DMF. The crude material was dissolved in CH_2_Cl_2_ (100 mL) and hexane (200 mL) added. The CH_2_Cl_2_ was removed *in vacuo* and the resulting precipitate filtered and dried to yield the product as an off-white solid (128 mg, 0.08 mmol, 64%). **IR** (ν_max_ / cm^-1^) 2953, 2919, 2852, 2362, 1598, 1449, 1362, 1247, 905, 731; **^1^H NMR** (400 MHz, CDCl_3_) δ_H_ 7.66 (s, 3H, **H_e_**), 7.54 (s, 6H, **H_f_**), 7.43 (s, 3H, **H_b_**), 7.35 (s, 3H, **H_g_**), 7.15 (s, 6H, **H_c_**), 5.55 (s, 6H, **H_d_**), 3.98 (s, 12H, **H_h_**), 3.86 (s, 12H, **H_i_**), 2.41 (s, 18H, **H_j_**), 1.31 (s, 54H, **H_a_**); **^13^C NMR** (126 MHz, CDCl_3_) δ_C_ 152.0, 148.2, 141.7, 136.0, 135.1, 133.9, 130.1, 123.7, 123.6, 123.0, 122.5, 119.6, 55.0, 54.6, 49.5, 35.1, 31.5, 15.8; **HRMS** (ESI+) calc. for C_99_H_129_N_15_ [M+H]^+^ 1529.0634; found [M+H]^+^ 1529.0630.

**Figure S117** - ^1^H NMR (CDCl_3_) spectrum of **A3**.

**Figure S118** - ^1^-^1^H COSY NMR (CDCl_3_) spectrum of **A3**.

**Figure S119** - ^13^C NMR (CDCl_3_) spectrum of **A3**.

**Figure S120** - ^1^H-^13^C HSQC NMR (CDCl_3_) spectrum of **A3**.

**Figure S121** - ^1^H-^13^C HMBC NMR (CDCl_3_) spectrum of **A3**.

**Figure S122** - IR spectrum of **A3**.

**Figure S123** - HRMS spectrum of **A3**.

**Figure S124** - TGA trace with plotted first derivative of **A3**.

**Figure S125** - DSC traces and corresponding TGA curve (black) of **A3** subjected to heat-cool-heat-cool (HCHC) cycles to 150 °C. DSC traces: Initial upscan shown as a solid red line, downscan as a blue line, second upscan as a dashed red line and second downscan as a dashed blue line.

**Figure S126** - Optical images of desolvated **A3** at different stages subjected to HCHC cycles to 200 °C.

**Figure S127** – ^1^H NMR (CDCl_3_) of **A3** post DSC.

**Figure S128** – CO_2_ adsorption (filled) and desorption (empty) isotherms for **A3**.

**Figure S129** - Stacked PXRDs of **A3**; solvated (black), desolvated (blue), post-sorption (green) and post DSC to 200 °C (orange).

## S2.4. Synthesis of Cage[*n*]Rotaxanes

*Note:* Stock solutions (5 mg/mL) of tetrakis(acetonitrile)copper(I) hexafluorophosphate ([Cu(MeCN)_4_]PF_6_) (50 mg) in CH_2_Cl_2_ (10 mL) were made before dispensing the correct amount into the reaction mixture. Henceforth, this stock solution will be referred to as **solution 1**.

Cage[2]Rotaxane, **CR1**

To a vial containing **M** (68 mg, 0.14 mmol, 1.0 eq.), **C1** (104 mg, 0.14 mmol, 1 eq.), **S** (34 mg, 0.14 mmol, 1 eq.) and *N*,*N*-diisopropylethylamine (44 µL, 0.27 mmol, 2 eq.) under N_2_ in EtOH:CH_2_Cl_2_ (1:1 v/v, 12 mL), was added **solution 1** (1 mL, [Cu(MeCN)_4_]PF_6_ (0.014 mmol, 0.1 eq.)), and the mixture stirred at RT for 3 days. The solvent was removed *in vacuo* and to the residue, CH_2_Cl_2_ (10 mL) was added, and the mixture washed with EDTA-NH_3_ solution (80 mL). The aqueous phase was extracted with CH_2_Cl_2_ (3 x 50 mL). The organic phases were collected, dried (MgSO_4_), filtered and concentrated *in vacuo*. The crude material was purified by column chromatography, first ((SiO_2_ deactivated with NEt_3_) CH_2_Cl_2_:EtOAc (1:1)) to elute residual azide and macrocycle, and then ((SiO_2_ deactivated with NEt_3_) 10% MeOH in CH_2_Cl_2_:EtOAc (1:1)) to elute the rotaxane species. The crude rotaxane species was purified by RPTLC (C8 SiO_2_, deactivated with NEt_3_) 2% NEt_3_ in MeCN, 6 runs) to yield the product as a colourless solid (20 mg, 0.014 mmol, 10%).

**IR** (ν_max_ / cm^-1^) 2948, 2922, 2855, 2358, 2339, 1728, 1608, 1510, 1454, 1249, 1098, 785; **^1^H NMR** (400 MHz, CDCl_3_) δ_H_ 9.67 (s, 1H, **H_e_**), 7.77 (t, J = 7.7 Hz, 2H, **H_H_**), 7.71 (d, J = 7.5 Hz, 2H, **H_G_**), 7.43 (d, J = 7.7 Hz, 2H, **H_I_**), 7.36 (s, 2H, **H_f_**), 7.32 (s, 1H, **H_b_**), 7.29 (s, 1H, **H_g_**), 7.19 (t, J = 7.5 Hz, 2H, **H_m_**), 7.08-7.00 (m, 8H, **H_c,l,n_**), 6.56 – 6.45 (m, 8H, **H_C,D_**), 4.83 (s, 2H, **H_d_**), 4.72 (d, J = 8.3 Hz, 2H, two of **H_B_**), 4.42 (d, J = 6.1 Hz, 2H, two of **H_B_**), 4.28 (q, J = 12.1 Hz, 4H, **H_F_**), 3.95 (s, 8H, **H_k_**), 3.85 (s, 8H, **H_j_**), 3.81 – 3.66 (m, 8H, **H_E_** and **H_h_**), 3.61 (s, 4H, **H_i_**), 2.40 (s, 6H, **H_o_**), 2.38 (s, 12H, **H_p_**), 2.16 (m, 4H, **H_A_**), 1.15 (s, 18H, **H_a_**); **Diffusion coefficient** (500 MHz, CDCl_3_) *D*: 9.46 × 10^-10^ m^2^s^-1^; **^13^C NMR** (101 MHz, CDCl_3_) δ_C_; 159.8, 159.1, 155.4, 151.2, 146.1, 141.2, 139.8, 137.3, 136.0, 135.9, 135.2, 135.0, 134.3, 130.4, 129.3, 127.7, 125.9, 124.3, 123.9, 123.6, 123.3, 121.4, 121.0, 119.9, 115.1, 73.0, 70.2, 66.4, 54.7, 54.1, 49.6, 46.3, 34.8, 31.5, 29.9, 25.0, 15.8; **HRMS** (ESI+) calc. for C_95_H_113_N_11_O_4_ [M+H]^+^ 1472.9055; found [M+H]^+^ 1472.9128.

**Figure S130** - ^1^H NMR (CDCl_3_) spectrum of **CR1**.

**Figure S131** - ^1^H-^1^H COSY NMR (CDCl_3_) spectrum of **CR1**.

**Figure S132** - ^13^C NMR (CDCl_3_) spectrum of **CR1**.

**Figure S133** - ^1^H-^13^C HSQC NMR (CDCl_3_) spectrum of **CR1**.

**Figure S134** - ^1^H-^13^C HMBC NMR (CDCl_3_) spectrum of **CR1**.

**Figure S135** - DOSY NMR (CDCl_3_) spectrum of **CR1**.

**Figure S136** - Representative diffusion decay curve (left) and fit (right) for **CR1**.

**Table S1** – Chemical shifts and diffusion coefficients of peaks for **CR1**. ^a^Error shown is standard deviation for diffusion coefficients.

| **Chemical Shift (ppm)** | **Diffusion Coefficient (m^2^s^-1^)** |
| --- | --- |
| 9.694 | 9.68 × 10^-10^ |
| 7.812 | 9.6 × 10^-10^ |
| 7.796 | 9.6 × 10^-10^ |
| 7.781 | 9.63 × 10^-10^ |
| 7.741 | 9.61 × 10^-10^ |
| 7.726 | 9.51 × 10^-10^ |
| 7.466 | 9.53 × 10^-10^ |
| 7.451 | 9.47 × 10^-10^ |
| 7.387 | 9.48 × 10^-10^ |
| 7.315 | 9.32 × 10^-10^ |
| 7.287 | 3.79 × 10^-10^ |
| 7.234 | 9.8 × 10^-10^ |
| 7.219 | 9.62 × 10^-10^ |
| 7.204 | 9.57 × 10^-10^ |
| 7.104 | 9.54 × 10^-10^ |
| 7.089 | 9.49 × 10^-10^ |
| 6.578 | 9.66 × 10^-10^ |
| 6.561 | 9.58 × 10^-10^ |
| 6.519 | 9.52 × 10^-10^ |
| 6.502 | 9.42 × 10^-10^ |
| 4.858 | 9.47 × 10^-10^ |
| 4.751 | 9.44 × 10^-10^ |
| 4.735 | 8.92 × 10^-10^ |
| 4.455 | 8.49 × 10^-10^ |
| 4.443 | 8.79 × 10^-10^ |
| 4.347 | 9.52 × 10^-10^ |
| 4.323 | 9.55 × 10^-10^ |
| 4.297 | 9.36 × 10^-10^ |
| 4.272 | 9.44 × 10^-10^ |
| 3.972 | 9.49 × 10^-10^ |
| 3.877 | 9.58 × 10^-10^ |
| 3.732 | 9.42 × 10^-10^ |
| 3.828 | 9.56 × 10^-10^ |
| 3.801 | 9.58 × 10^-10^ |
| 3.771 | 9.44 × 10^-10^ |
| 3.745 | 9.31 × 10^-10^ |
| 3.635 | 9.42 × 10^-10^ |
| 2.429 | 9.5 × 10^-10^ |
| 2.41 | 9.5 × 10^-10^ |
| 1.18 | 9.62 × 10^-10^ |
| **Average Diffusion Coefficient (m^2^s^-1^)** | 9.46 × 10^-10^ ± 3.22 × 10^-12 a^ |

**Figure S137** - IR spectrum of **CR1**.

**Figure S138** - HRMS spectrum of **CR1**.

**Figure S139** - TGA trace with plotted first derivative of **CR1**.

**Figure S140** - DSC traces and corresponding TGA curve (black) of **CR1** subjected to heat-cool-heat-cool (HCHC) cycles to 140 °C. DSC traces: Initial upscan shown as a solid red line, downscan as a blue line, second upscan as a dashed red line and second downscan as a dashed blue line.

**Figure S141** - Optical images of desolvated **CR1** at different stages subjected to HCHC cycles to 140 °C.

**Figure S142** - ^1^H NMR (CDCl_3_) spectrum of **CR1** post DSC.

**Figure S143** - Stacked PXRDs of **CR1**; solvated (black), desolvated (blue) and post DSC to 140 °C (orange).

Cage[3]Rotaxane, **CR2**

To a vial containing **M** (87 mg, 0.18 mmol, 2.0 eq.), **C2** (69 mg, 0.09 mmol, 1 eq.), **S** (44 mg, 0.18 mmol, 2 eq.) and *N*,*N*-diisopropylethylamine (30 µL, 0.18 mmol, 2 eq.) under N_2_ in EtOH:CH_2_Cl_2_ (1:1 v/v, 12 mL) was added **solution 1** (1.35 mL, [Cu(MeCN)_4_]PF_6_ (0.018 mmol, 0.2 eq)) and stirred at RT for 3 days. The solvent was removed *in vacuo* and to the residue, CH_2_Cl_2_ (10 mL) was added, and the mixture washed with EDTA-NH_3_ solution (80 mL). The aqueous phase was extracted with CH_2_Cl_2_ (3 x 50 mL). The organic phases were collected, dried (MgSO_4_), filtered and concentrated *in vacuo*. The crude material was purified by column chromatography, first ((SiO_2_ deactivated with NEt_3_) CH_2_Cl_2_:EtOAc (1:1)) to elute residual azide and macrocycle, and then ((SiO_2_ deactivated with NEt_3_) 10% MeOH in CH_2_Cl_2_:EtOAc (1:1)) to elute the rotaxane species. The crude rotaxane species was purified by RPTLC (C8 SiO_2_, deactivated with NEt_3_) 2% NEt_3_ in MeCN, 13 runs) to yield the product as a light pink solid (25 mg, 0.01 mmol, 13%).

**IR** (ν_max_ / cm^-1^) 2952, 2859, 2115, 2070, 1575, 1505, 1243, 1077, 993, 785; **^1^H NMR** (500 MHz, CDCl_3_) δ 9.65 (s, 2H, **H_e_**), 7.76 (t, J = 7.8 Hz, 4H, **H_H_**), 7.70 (d, J = 7.2 Hz, 4H, **H_G_**), 7.43 (d, J = 7.6 Hz, 4H, **H_I_**), 7.31 (s, 2H, **H_b_**), 7.27 (s, 4H, **H_f_**), 7.19 – 7.13 (m, 2H, **H_l_**), 7.06 – 7.04 (m, 2H, **H_m,n_**), 7.03 (s, 4H, **H_c_**), 6.99 (s, 2H, **H_g_**), 6.56 – 6.46 (m, 16H, **H_C_**_,_**_D_**), 4.81 (s, 4H, **H_d_**), 4.69 (q, J = 7.2 Hz, 4H, two of **H_B_**), 4.43 (q, J = 6.7 Hz, 4H, two of **H_B_**), 4.28 (dd, J = 28.5, 12.1 Hz 8H, **H_F_**), 3.92 (s, 4H, **H_j_**), 3.84 (s, 4H, **H_k_**), 3.75 (m, 8H, **H_E_**), 3.70 (s, 8H, **H_h_**), 3.60 (dd, J = 34.1, 20.5 Hz, 8H, **H_i_**), 2.37 (s, 12H, **H_o_**), 2.36 (s, 6H, **H_p_**), 2.16 (t, J = 6.0 Hz, 8H, **H_A_**), 1.15 (s, 36H, **H_a_**); **Diffusion coefficient** (500 MHz, CDCl_3_) *D*: 1.16 × 10^-9^ m^2^s^-1^; **^13^C NMR** (126 MHz, CDCl_3_) δ_C_ 159.8, 159.1, 155.4, 151.1, 146.1, 141.0, 139.8, 137.3, 136.0, 135.9, 135.1, 134.9, 134.3, 130.4, 129.3, 127.9, 127.7, 125.8, 124.2, 123.9, 123.6, 123.3, 122.5, 121.3, 120.9, 119.9, 115.1, 73.0, 70.2, 66.4, 54.7, 54.5, 54.1, 53.6, 49.6, 49.5, 46.2, 34.8, 31.5, 25.0, 15.7; **HRMS** (ESI+) calc. for C_142_H_166_N_16_O_8_ [M+H]^+^ 2224.3153 and [M+2H]^2+^ 1112.6616; found [M+2H]^2+^ 1112.6631.

Figure **S144** - ^1^H NMR (CDCl_3_) spectrum of **CR2**.

Figure **S145** - ^1^H-^1^H COSY NMR (CDCl_3_) spectrum of **CR2**.

Figure **S146** - ^13^C NMR (CDCl_3_) spectrum of **CR2**.

Figure **S147** - ^1^H-^13^C HSQC NMR (CDCl_3_) spectrum of **CR2**.

Figure **S148** - ^1^H-^13^C HMBC NMR (CDCl_3_) spectrum of **CR2**.

**Figure S149** - DOSY NMR (CDCl_3_) spectrum of **CR2**.

**Figure S150** – Representative diffusion decay curve (left) and fit (right) for **CR2**.

**Table S2** - Chemical shifts and diffusion coefficients of peaks for **CR2**. ^a^Error shown is standard deviation for diffusion coefficients.

| **Chemical Shift (ppm)** | **Diffusion Coefficient (m^2^s^-1^)** |
| --- | --- |
| 9.67 | 1.15 × 10^-9^ |
| 7.785 | 1.19 × 10^-9^ |
| 7.77 | 1.17 × 10^-9^ |
| 7.731 | 1.17 × 10^-9^ |
| 7.716 | 1.14 × 10^-9^ |
| 7.455 | 1.18 × 10^-9^ |
| 7.44 | 1.15 × 10^-9^ |
| 7.286 | 3.79 × 10^-9^ |
| 7.048 | 1.17 × 10^-9^ |
| 6.57 | 1.21 × 10^-9^ |
| 6.554 | 1.15 × 10^-9^ |
| 6.509 | 1.18 × 10^-9^ |
| 6.493 | 1.13 × 10^-9^ |
| 4.835 | 1.14 × 10^-9^ |
| 4.303 | 1.22 × 10^-9^ |
| 4.293 | 1.14 × 10^-9^ |
| 3.943 | 1.14 × 10^-9^ |
| 3.863 | 1.15 × 10^-9^ |
| 3.817 | 1.18 × 10^-9^ |
| 3.792 | 1.16 × 10^-9^ |
| 3.764 | 1.15 × 10^-9^ |
| 3.738 | 1.18 × 10^-9^ |
| 3.717 | 1.14 × 10^-9^ |
| 3.629 | 1.23 × 10^-9^ |
| 3.615 | 1.15 × 10^-9^ |
| 2.387 | 1.17 × 10^-9^ |
| 1.172 | 1.16 × 10^-9^ |
| 7.084 | 1.19 × 10^-9^ |
| 7.023 | 1.14 × 10^-9^ |
| 7.333 | 1.15 × 10^-9^ |
| 4.727 | 1.18 × 10^-9^ |
| 4.711 | 1.15 × 10^-9^ |
| 4.446 | 1.14 × 10^-9^ |
| 4.441 | 1.13 × 10^-9^ |
| **Average Diffusion Coefficient (m^2^s^-1^)** | 1.16 × 10^-9^ ± 5.78 × 10^-12 a^ |

**Figure S151** - IR spectrum of **CR2**.

**Figure S152** - HRMS spectrum of **CR2**.

**Figure S153** - TGA trace with plotted first derivative of **CR2**.

**Figure S154** - DSC traces and corresponding TGA curve (black) of **CR2** subjected to heat-cool-heat-cool (HCHC) cycles to 160 °C. DSC traces: Initial upscan shown as a solid red line, downscan as a blue line, second upscan as a dashed red line and second downscan as a dashed blue line.

**Figure S155** - Optical images of desolvated **CR2** at different stages subjected to HCHC cycles to 160 °C.

**Figure S156** - ^1^H NMR (CDCl_3_) spectrum of **CR2** post DSC.

**Figure S157** - Stacked PXRDs of **CR2**; solvated (black), desolvated (blue) and post DSC to 160 °C (orange).

Cage[4]Rotaxane, **CR3**

To a vial containing **M** (43 mg, 0.09 mmol, 3.0 eq.), **C3** (24 mg, 0.01 mmol, 1 eq.), **S** (22 mg, 0.03 mmol, 3 eq.) and *N,N*-diisopropylethylamine (10 µL, 0.02 mmol, 2 eq.) under N_2_ in EtOH:CH_2_Cl_2_ (1:1 v/v, 10 mL) was added **solution 1** (0.6 mL, [Cu(MeCN)_4_]PF_6_ (0.009 mmol, 0.3 eq)) and the mixture stirred at RT for 3 days. The solvent was removed *in vacuo* and to the residue, CH_2_Cl_2_ (10 mL) was added, and the mixture washed with EDTA-NH_3_ solution (80 mL). The aqueous phase was extracted with CH_2_Cl_2_ (3 x 50 mL). The organic phases were collected, dried (MgSO_4_), filtered and concentrated *in vacuo*. The crude material was purified by column chromatography, first ((SiO_2_ deactivated with NEt_3_) CH_2_Cl_2_:EtOAc (1:1)) to elute residual azide and macrocycle, and then ((SiO_2_ deactivated with NEt_3_) 10% MeOH in CH_2_Cl_2_:EtOAc (1:1)) to elute the rotaxane species. The crude rotaxane species was purified by RPTLC (C8 SiO_2_, deactivated with NEt_3_) 2% NEt_3_ in MeCN, 16 runs) to yield the product as a white solid (10 mg, 0.003 mmol, 30%).

**IR** (ν_max_ / cm^-1^) 3067, 2952, 2867, 2359, 2333, 1606, 1511, 1248, 1097, 993, 786; **^1^H NMR** (400 MHz, CDCl_3_) δ 9.64 (s, 3H, **H_e_**), 7.75 (t, J = 7.7 Hz, 6H, **H_H_**), 7.69 (d, J = 7.6 Hz, 6H, **H_G_**), 7.41 (d, J = 7.6 Hz, 6H, **H_I_**), 7.29 (s, 3H, **H_b_**), 7.00 - 6.98 (m, 15H, **H_c_**_,_**_f_**_,_**_g_**), 6.54 (d, J = 8.7 Hz, 12H, **H_D_**), 6.47 (d, J = 8.7 Hz, 12H, **H_C_**), 4.78 (s, 6H, **H_d_**), 4.66 (d, J = 8.0 Hz, 6H, two of **H_B_**), 4.44 (d, J = 7.7 Hz, 6H, two of **H_B_**), 4.27 (s, 12H, **H_F_**), 3.79 – 3.70 (m, 12H, **H_E_**), 3.68 (s, 12H, **H_h_**), 3.59 (s, 12H, **H_i_**), 2.34 (s, 18H, **H_j_**), 2.17 (d, J = 6.6 Hz, 12H, **H_A_**), 1.14 (s, 54H, **H_a_**); **Diffusion coefficient** (500 MHz, CDCl_3_) *D*: 7.60 × 10^-10^ m^2^s^-1^; **^13^C NMR** (126 MHz, CDCl_3_) δ 159.8, 159.1, 155.4, 151.1, 146.1, 139.9, 137.2, 135.9, 135.0, 134.2, 130.4, 129.3, 127.9, 124.2, 124.0, 123.5, 123.2, 121.1, 120.9, 119.8, 115.1, 73.0, 70.2, 66.4, 54.5, 54.0, 49.6, 46.4, 34.8, 31.5, 25.0, 15.7. **HRMS** (ESI+) calc. for C_189_H_219_N_21_O_12_ [M+H]^+^ 2975.7250, [M+2H]^2+^ 1488.8703 and [M+3H]^3+^ 992.9162; found [M+2H]^2+^ 1488.8698 and [M+3H]^3+^ 992.9128.

**Figure S158** - ^1^H NMR (CDCl_3_) spectrum of **CR3**.

**Figure S159** - ^1^H-^1^H COSY NMR (CDCl_3_) spectrum of **CR3**.

**Figure S160** - ^13^C NMR (CDCl_3_) spectrum of **CR3**.

**Figure S161** - ^1^H-^13^C HSQC NMR (CDCl_3_) spectrum of **CR3**.

**Figure S162** - ^1^H-^13^C HMBC NMR (CDCl_3_) spectrum of **CR3**.

**Figure S163** - DOSY NMR (CDCl_3_) spectrum of **CR3**.

**Figure S164** – Representative diffusion decay curve (left) and fit (right) for **CR3**.

**Table S3** - Chemical shifts and diffusion coefficients of peaks for **CR3**. ^a^Error shown is standard deviation for diffusion coefficients.

| **Chemical Shift (ppm)** | **Diffusion Coefficient (m^2^s^-1^)** |
| --- | --- |
| 7.769 | 8.15 × 10^-10^ |
| 7.754 | 7.8 × 10^-10^ |
| 7.716 | 7.6 × 10^-10^ |
| 7.7 | 7.75 × 10^-10^ |
| 7.282 | 3.57 × 10^-9^ |
| 7.026 | 7.77 × 10^-10^ |
| 6.564 | 7.99 × 10^-10^ |
| 6.548 | 7.81 × 10^-10^ |
| 6.498 | 7.5 × 10^-10^ |
| 6.481 | 7.42 × 10^-10^ |
| 4.798 | 7.21 × 10^-10^ |
| 4.285 | 7.51 × 10^-10^ |
| 3.809 | 8.36 × 10^-10^ |
| 3.782 | 7.89 × 10^-10^ |
| 3.759 | 7.62 × 10^-10^ |
| 3.733 | 7.75 × 10^-10^ |
| 3.701 | 7.31 × 10^-10^ |
| 3.608 | 7.37 × 10^-10^ |
| 2.358 | 7.53 × 10^-10^ |
| 1.159 | 7.53 × 10^-10^ |
| 9.64 | 7.32 × 10^-10^ |
| 4.689 | 7.87 × 10^-10^ |
| 4.672 | 7.1 × 10^-10^ |
| 4.458 | 7.46 × 10^-10^ |
| 4.445 | 6.83 × 10^-10^ |
| **Average Diffusion Coefficient (m^2^s^-1^)** | 7.60 × 10^-10^ ± 3.36 × 10^-11^ |

**Figure S165** IR spectrum of **CR3**.

**Figure S166** - HRMS spectrum of **CR3**.

**Figure S167** - TGA trace with plotted first derivative of **CR3**.

**Figure S168** - DSC traces and corresponding TGA curve (black) of **CR3** subjected to heat-cool-heat-cool (HCHC) cycles to 150 °C. DSC traces: Initial upscan shown as a solid red line, downscan as a blue line, second upscan as a dashed red line and second downscan as a dashed blue line.

**Figure S169** - Optical images of desolvated **CR3** at different stages subjected to HCHC cycles to 150 °C.

**Figure S170** - ^1^H NMR (CDCl_3_) spectrum of **CR3** post DSC.

**Figure S171** - Stacked PXRDs of **CR3**; solvated (black), desolvated (blue) and post DSC to 150 °C (orange).

## S2.5. Synthesis of Cage[*n*]Rotaxane Bulk Species

*Note:* Stock solutions (5 mg/mL) of tetrakis(acetonitrile)copper(I) hexafluorophosphate ([Cu(MeCN)_4_]PF_6_) (50 mg) in CH_2_Cl_2_ (10 mL) were made before dispensing the correct amount into the reaction mixture. As stated previously, this is referred to as **solution 1**.

Cage[2]Rotaxane Bulk Species, **BS1**

To a vial containing **M** (136 mg, 0.28 mmol, 1.0 eq.), **C1** (210 mg, 0.28 mmol, 1 eq.), **S** (69 mg, 0.28 mmol, 1 eq.) and *N*,*N*-diisopropylethylamine (93 µL, 0.56 mmol, 2 eq.) under N_2_ in EtOH:CH_2_Cl_2_ (1:1 v/v, 25 mL) was added **solution 1** (2.2 mL, [Cu(MeCN)_4_]PF_6_ (0.028 mmol, 0.1 eq)) and the mixture stirred at RT for 3 days. The solvent was removed *in vacuo* and to the residue, CH_2_Cl_2_ (10 mL) was added, and the mixture washed with EDTA-NH_3_ solution (50 mL). The aqueous phase was extracted with CH_2_Cl_2_ (3 x 50 mL). The organic phases were collected, dried (MgSO_4_), filtered and concentrated *in vacuo* to yield the crude product, which was purified by column chromatography, first ((SiO_2_ deactivated with NEt_3_) CH_2_Cl_2_:EtOAc (1:1)) to elute residual azide and macrocycle, followed by ((SiO_2_ deactivated with NEt_3_) 10% MeOH in CH_2_Cl_2_:EtOAc (1:1)) to elute the rotaxane species as an off-white solid (143 mg); **IR** (ν_max_ / cm^-1^) 3318, 2953, 2905, 2861, 2373, 1656, 1605, 1444, 1243, 1093, 784, 703; **HRMS** (ESI+) found [M+H]^+^ 1472.9105 (**A**), [M+2H]^2+^ 736.9536 (**A**), [M+H]^+^ 990.6966 (**B**); **Ratio** of target rotaxane *vs* non-interlocked axle; **A** : **B** = 5.88 : 1; **BET SA** = 4.984 m^2^g^-1^.

**Figure S172** - ^1^H NMR (CDCl_3_) spectrum of **BS1**.

**Figure S173** - Zoomed in ^1^H NMR (CDCl_3_) spectrum of **BS1** showing integration of triazole peak on rotaxane **A** (**H_A_**) *vs* integration of methylene peak of non-interlocked axle **B** (**H_B_**).

**Figure S174** - IR spectrum of **BS1**.

**Figure S175** - HRMS spectrum of **BS1**.

**Figure S176** - TGA trace with plotted first derivative of **BS1**.

**Figure S177** - CO_2_ adsorption (filled) and desorption (empty) isotherms for **BS1**.

**Figure S178** – N_2_ adsorption (filled) and desorption (empty) isotherms for **BS1**.

**Figure S179** – BET surface area plot of **BS1**.

**Figure S180** - DSC traces and corresponding TGA curve (black) of **BS1** subjected to heat-cool-heat-cool (HCHC) cycles to 150 °C. DSC traces: Initial upscan shown as a solid red line, downscan as a blue line, second upscan as a dashed red line and second downscan as a dashed blue line.

**Figure S181** - Optical images of desolvated **BS1** at different stages subjected to HCHC cycles to 150 °C.

**Figure S182** - ^1^H NMR (CDCl_3_) of **BS1** post HC DSC.

**Figure S183** - Stacked PXRDs of **BS1**; solvated (black), desolvated (blue), post-sorption (green) and post DSC to 150 °C (orange).

Cage[3]Rotaxane Bulk Species, **BS2**

To a vial containing **M** (135 mg, 0.28 mmol, 2.0 eq.), **C2** (105 mg, 0.14 mmol, 1 eq.), **S** (69 mg, 0.28 mmol, 2 eq.) and *N*,*N*-diisopropylethylamine (46 µL, 0.28 mmol, 2 eq.) under N_2_ in EtOH:CH_2_Cl_2_ (1:1 v/v, 20 mL) was added **solution 1** (2 mL, [Cu(MeCN)_4_]PF_6_ (0.028 mmol, 0.2 eq)) and the mixture stirred at RT for 3 days. The solvent was removed *in vacuo* and to the residue, CH_2_Cl_2_ (10 mL) was added, and the mixture washed with EDTA-NH_3_ solution (50 mL). The aqueous phase was extracted with CH_2_Cl_2_ (3 x 50 mL). The organic phases were collected, dried (MgSO_4_), filtered and concentrated *in vacuo* to yield the crude product, which was purified by column chromatography, first ((SiO_2_ deactivated with NEt_3_) CH_2_Cl_2_:EtOAc (1:1)) to elute residual azide and macrocycle, followed by ((SiO_2_ deactivated with NEt_3_) 10% MeOH in CH_2_Cl_2_:EtOAc (1:1)) to elute the rotaxane species as an off-white solid (111 mg); **IR** (ν_max_ / cm^-1^) 3280, 2948, 2908, 2863, 1654, 1596, 1447, 1322, 1146, 1115, 704, 619; **HRMS** (ESI+) found [M+2H]^2+^ 1112.6610 (**A**), [M+3H]^3+^ 742.2238 (**A**), [M+H]^+^ 1743.0804 (**B**), [M+2H]^2+^ 872.0519 (**B**), [M+3H]^3+^ 581.5750 (**B**); **Ratio** of rotaxane species *vs* non-interlocked axle; **A & B** : **C** = 12.5 : 1; **BET SA** = 12.818 m^2^g^-1^.

**Figure S184** - ^1^H NMR (CDCl_3_) spectrum of **BS2**.

**Figure S185** - Partial ^1^H NMR (CDCl_3_) NMR spectrum of **BM2** showing integration of rotaxane species triazole peak against axle methylene proton.

**Figure S186** - IR spectrum of **BS2**.

**Figure S187** - HRMS spectrum of **BS2**.

**Figure S188** - TGA trace and plotted first derivative of **BS2**.

**Figure S189** - CO_2_ adsorption (filled) and desorption (empty) isotherms for **BS2**.

**Figure S190** – N_2_ adsorption (filled) and desorption (empty) isotherms for **BS2**.

**Figure S191** – BET surface area plot of **BS2**.

**Figure S192** - DSC traces and corresponding TGA curve (black) of **BS2** subjected to heat-cool-heat-cool (HCHC) cycles to 150 °C. DSC traces: Initial upscan shown as a solid red line, downscan as a blue line, second upscan as a dashed red line and second downscan as a dashed blue line.

**Figure S193** - Optical images of desolvated **BS2** at different stages subjected to HCHC cycles to 150 °C.

**Figure S194** - ^1^H NMR (CDCl_3_) spectrum of **BS2** post DSC.

**Figure S195** - Stacked PXRDs of **BS2**; solvated (black), desolvated (blue), post-sorption (green) and post DSC to 150 °C (orange).

Cage[4]Rotaxane Bulk Species, **BS3**

To a vial containing **M** (135 mg, 0.17 mmol, 3.0 eq.), **C3** (135 mg, 0.17 mmol, 1 eq.), **S** (123 mg, 0.5 mmol, 3 eq.) and *N*,*N*-diisopropylethylamine (85 µL, 0.02 mmol, 2 eq.) under N_2_ in EtOH: CH_2_Cl_2_ (1:1 v/v, 30 mL) was added **solution 1** (3.8 mL, [Cu(MeCN)_4_]PF_6_ (0.05 mmol, 0.3 eq)) and the mixture stirred at RT for 3 days. The solvent was removed *in vacuo* and to the residue, CH_2_Cl_2_ (10 mL) was added, and the mixture washed with EDTA-NH_3_ solution (50 mL). The aqueous phase was extracted with CH_2_Cl_2_ (3 x 50 mL). The organic phases were collected, dried (MgSO_4_), filtered and concentrated *in vacuo* to yield the crude product, which was purified by column chromatography, first ((SiO_2_ deactivated with NEt_3_) CH_2_Cl_2_:EtOAc (1:1)) to elute residual azide and macrocycle, followed by ((SiO_2_ deactivated with NEt_3_) 10% MeOH in CH_2_Cl_2_:EtOAc (1:1)) to elute the rotaxane species as a light orange solid (310 mg); **IR** (ν_max_ / cm^-1^) 3071, 2950, 2900, 2858, 1607, 1507, 1449, 1243, 1075, 992, 840, 787; **HRMS** (ESI+) found [M+2H]^2+^ 1488.8680 (**A**), [M+3H]^3+^ 992.9146 (**A**), [M+2H]^2+^ 1248.2719 (**B**); **Ratio** of rotaxane species *vs* non-interlocked axle: No formation of non-interlocked axle observed; Ratio of **A** : **B** = 2.2 : 1. **BET SA** = 1.756 m^2^g^-1^.

**Figure S196** - ^1^H NMR (CDCl_3_) spectrum of **BS3**.

**Figure S197** – Partial stacked ^1^H NMR (CDCl_3_) spectra of rotaxane **CR3** (red), non-interlocked axle, **A3** and **BS3**.

**Figure S198** - Partial ^1^H NMR (CDCl_3_) NMR spectrum of **BM3** showing integration of individual triazole peaks of rotaxane species. Reference deconvolution applied: Linewidth of 4.6 Hz with a Lorentzian shape.

**Figure S199** - IR spectrum of **BS3**.

**Figure S200** - HRMS spectrum of **BS3**.

**Figure S201** - TGA trace and plotted first derivative of **BS3**.

**Figure S202** - CO_2_ adsorption (filled) and desorption (empty) isotherms for **BS3**.

**Figure S203** – N_2_ adsorption (filled) and desorption (empty) isotherms of **BS3**.

**Figure S204** – BET surface area plot of **BS3**.

**Figure S205** - DSC traces and corresponding TGA curve (black) of **BS3** subjected to heat-cool-heat-cool (HCHC) cycles to 200 °C. DSC traces: Initial upscan shown as a solid red line, downscan as a blue line, second upscan as a dashed red line and second downscan as a dashed blue line.

**Figure S206** - Optical images of desolvated **BS3** at different stages subjected to HCHC cycles to 200 °C.

**Figure S207** - ^1^H NMR (CDCl_3_) of **BS3** post HC DSC.

**Figure S208** - Stacked PXRDs of **BS3**; solvated (black), desolvated (blue), post-sorption (green) and post DSC to 200 °C (orange).

# S3: Solubility Measurements

**General Procedure:** Samples of **C1-C3**, **A1-A3**, **CR1-CR3** and **M** (10 mg) were placed in separate glass vials and the vial tared. CHCl_3_ (0.5-1.0% EtOH as stabiliser) was added in 20 μL increments *via* micro syringe and weighed by mass after each addition. Total solubilisation of the material was determined visually by the absence of any solid particles remaining in solution, and the weight of the total amount of CHCl_3_ recorded. The vial was then concentrated to dryness and re-weighed to ensure the same amount of material remained at the start. This was then repeated three times to obtain the results in **Table S1** after converting the weight of CHCl_3_ added to mL by using the density (1.492 g/mL at 25 °C) and dividing by the mass of the sample.

**Table S4** – Results of solubility measurements on cage samples.

| **Cage Species** | **Solubility_1_ (g/mL)** | **Solubility_2_ (g/mL)** | **Solubility_3_ (g/mL)** | **Avg. Solubility (g/mL)** | **Avg. Solubility (mol/L)** |
| --- | --- | --- | --- | --- | --- |
| **C1** | 0.020 | 0.025 | 0.021 | 0.02 ± 0.003 | 0.03 ± 0.004 |
| **C2** | 0.025 | 0.031 | 0.030 | 0.03 ± 0.003 | 0.04 ± 0.004 |
| **C3** | 0.055 | 0.053 | 0.055 | 0.05 ± 0.001 | 0.07 ± 0.001 |
| **A1** | 0.080 | 0.089 | 0.070 | 0.08 ± 0.010 | 0.08 ± 0.010 |
| **A2** | 0.120 | 0.100 | 0.100 | 0.11 ± 0.012 | 0.08 ± 0.009 |
| **A3** | 0.120 | 0.130 | 0.130 | 0.12 ± 0.006 | 0.08 ± 0.004 |
| **CR1** | 0.180 | 0.180 | 0.200 | 0.18 ± 0.012 | 0.12 ± 0.008 |
| **CR2** | 0.290 | 0.280 | 0.290 | 0.29 ± 0.006 | 0.13 ± 0.003 |
| **CR3** | 0.390 | 0.370 | 0.400 | 0.39 ± 0.015 | 0.13 ± 0.005 |
| **M** | 0.380 | 0.410 | 0.390 | 0.39 ± 0.015 | 0.82 ± 0.032 |

# S4: Computational Details

Models of the alkyne-functionalised cages **C1**, **C2** and **C3** were constructed in **Tri^2^Di^3^** topology, and subsequently modified with macrocycle and 3,5-di-*tert*-butylbenzyl azide as a stopper to obtain rotaxane structures (**CR1**, **CR2** and **CR3**, respectively) using the supramolecular toolkit (*stk*) software.^5^ All rotaxane structures were annealed with a molecular dynamics simulation in NVE ensemble for 10 ns, sampling 100 conformers along the simulation trajectory using a time step of 1 fs and a temperature of 700 K with OPLS4 force field as implemented in the Macromodel Suite.^6^ Each sampled conformer was further optimised with OPLS4 force field with a gradient convergence criterion of 0.05 kJ^-1^ A^-1^ with 2,500 maximum number of optimisation steps, using the Polak-Ribiere Conjugate Gradient minimisation algorithm.^7^ Subsequently, the lowest energy conformations were selected for analysis. The pore diameters were calculated with *pywindow* software (Table S5).^8^ The space fill representations (Figure S209) were illustrated with Mercury software.^9^

**Table S5** - Calculated pore diameters (using *pywindow* software) of the **A1**-**A3** and **CR1**-**CR3** lowest energy computational models.

| Cage/Rotaxane Species | Pore Diameter (Å) |
| --- | --- |
| A1 | 0.0 |
| A2 | 2.1 |
| A3 | 0.8 |
| CR1 | 0.0 |
| CR2 | 0.1 |
| CR3 | 0.4 |

**Figure S209** - Space fill representation of energy minimised molecular models for a-b) **CR1** c-d) **CR2** and e-f) **CR3**, where macrocycles are coloured in orange, cages are coloured in purple and stoppers are coloured in teal.

To map the electrostatic potentials (Figure S210), lowest energy conformations from molecular dynamics simulations for **C1-C3** and **CR1-CR3** were used for to single point energy calculations with the CP2K plane-wave density functional theory (DFT package),^10^ using the PBE functional,^11^ def2-TZVP basis set,^12^ a grid size of 400 Ry and a relative cut-off value of 100 Ry. Non-bonded interactions were accounted using the Grimme-D3 dispersion correction.^13^ The total electron density was coloured by the electrostatic potential, using ChimeraX software,^14^ with a surface offset value of 1.8 Å.

**Figure S210** - Electrostatic potential maps of a-c) **A1-A3** molecules d-f) **CR1-CR3** molecules. For clarity, all atoms are shown in ball stick configuration. The red and blue surfaces represent negative and positive regions of the potential, with 0.01 a.u step size for each elementary colour in the rainbow spectrum, green colour midpoint representing 0 kJ mol^-1^. The electrostatic maps are shown with 40% transparency to show atoms corresponding to the molecular structure clearly.

# S5: References

1.	R. L. Greenaway, V. Santolini, M. J. Bennison, B. M. Alston, C. J. Pugh, M. A. Little, M. Miklitz, E. G. B. Eden-Rump, R. Clowes, A. Shakil, H. J. Cuthbertson, H. Armstrong, M. E. Briggs, K. E. Jelfs and A. I. Cooper, *Nat. Commun.*, 2018, **9**, 2849.

2.	G. Ragazzon, A. Credi and B. Colasson, *Chem. Eur. J.*, 2017, **23**, 2149-2156.

3.	J. E. M. Lewis, R. J. Bordoli, M. Denis, C. J. Fletcher, M. Galli, E. A. Neal, E. M. Rochette and S. M. Goldup, *Chem. Sci.*, 2016, **7**, 3154-3161.

4.	H. Lahlali, K. Jobe, M. Watkinson and S. M. Goldup, *Angew. Chem. Int. Ed.*, 2011, **50**, 4151-4155.

5.	L. Turcani, E. Berardo and K. E. Jelfs, *J. Comput. Chem.*, 2018, **39**, 1931-1942.

6.	C. Lu, C. Wu, D. Ghoreishi, W. Chen, L. Wang, W. Damm, G. A. Ross, M. K. Dahlgren, E. Russell, C. D. Von Bargen, R. Abel, R. A. Friesner and E. D. Harder, *J. Chem. Theory Comput.*, 2021, **17**, 4291-4300.

7.	E. Polak and G. Ribiere, *Rev. Fr. Inform. Rech. Opérationnelle Sér. Rouge.*, 1969, **3**, 35-43.

8.	M. Miklitz and K. E. Jelfs, *J. Chem. Inf. Model.*, 2018, **58**, 2387-2391.

9.	C. F. Macrae, I. Sovago, S. J. Cottrell, P. T. A. Galek, P. McCabe, E. Pidcock, M. Platings, G. P. Shields, J. S. Stevens, M. Towler and P. A. Wood, *J. Appl. Crystallogr.*, 2020, **53**, 226-235.

10.	T. D. Kühne, M. Iannuzzi, M. Del Ben, V. V. Rybkin, P. Seewald, F. Stein, T. Laino, R. Z. Khaliullin, O. Schütt, F. Schiffmann, D. Golze, J. Wilhelm, S. Chulkov, M. H. Bani-Hashemian, V. Weber, U. Borštnik, M. Taillefumier, A. S. Jakobovits, A. Lazzaro, H. Pabst, T. Müller, R. Schade, M. Guidon, S. Andermatt, N. Holmberg, G. K. Schenter, A. Hehn, A. Bussy, F. Belleflamme, G. Tabacchi, A. Glöß, M. Lass, I. Bethune, C. J. Mundy, C. Plessl, M. Watkins, J. VandeVondele, M. Krack and J. Hutter, *J. Chem. Phys.*, 2020, **152**.

11.	J. P. Perdew, K. Burke and M. Ernzerhof, *Phys. Rev. Lett.*, 1996, **77**, 3865-3868.

12.	J. VandeVondele and J. Hutter, *J. Chem. Phys.*, 2007, **127**.

13.	S. Grimme, J. Antony, S. Ehrlich and H. Krieg, *J. Chem. Phys.*, 2010, **132**.

14.	E. F. Pettersen, T. D. Goddard, C. C. Huang, E. C. Meng, G. S. Couch, T. I. Croll, J. H. Morris and T. E. Ferrin, *Protein Sci.*, 2021, **30**, 70-82.
